# Enhanced quantification of metabolic activity for individual adipocytes by label-free FLIM

**DOI:** 10.1038/s41598-018-27093-x

**Published:** 2018-06-08

**Authors:** Michael Evers, Nunciada Salma, Sam Osseiran, Malte Casper, Reginald Birngruber, Conor L. Evans, Dieter Manstein

**Affiliations:** 1 Cutaneous Biology Research Center, Department of Dermatology, Massachusetts General Hospital, Harvard Medical School, Boston, MA 02114 USA; 2 Institute of Biomedical Optics, University of Lübeck, Lübeck, 23562 Germany; 3 Wellman Center for Photomedicine, Department of Dermatology, Massachusetts General Hospital, Harvard Medical School, Boston, MA 02114 USA

## Abstract

Fluorescence lifetime imaging microscopy (FLIM) of intrinsic fluorophores such as nicotinamide adenine dinucleotide (NADH) allows for label-free quantification of metabolic activity of individual cells over time and in response to various stimuli, which is not feasible using traditional methods due to their destructive nature and lack of spatial information. This study uses FLIM to measure pharmacologically induced metabolic changes that occur during the browning of white fat. Adipocyte browning increases energy expenditure, making it a desirable prospect for treating obesity and related disorders. Expanding from the traditional two-lifetime model of NADH to a four-lifetime model using exponential fitting and phasor analysis of the fluorescence decay results in superior metabolic assessment compared to traditional FLIM analysis. The four lifetime components can also be mapped to specific cellular compartments to create a novel optical ratio that quantitatively reflects changes in mitochondrial and cytosolic NADH concentrations and binding states. This widely applicable approach constitutes a powerful tool for studies where monitoring cellular metabolism is of key interest.

## Introduction

The demand for methods evaluating adipose tissue metabolism is growing rapidly, since obesity related diseases such as hypertension, type 2 diabetes, and many forms of cancer are increasing drastically^1,2^. In the human body, there are at least three types of fat cells with distinctive specialized metabolic functions. Excessively accumulated fat is known as white adipose tissue (WAT), whose main function is to store excess energy^3^. White adipocytes have few, relatively large lipid droplets and contain limited mitochondria. In contrast, brown adipose tissue (BAT) is specialized to dissipate energy as heat through nonshivering thermogenesis^4^. Brown adipocytes contain multilocular, relatively small lipid droplets and many mitochondria that contain the BAT-specific protein known as uncoupling protein 1 (UCP1). UCP1 plays an important role in increasing energy expenditure through uncoupling of oxidative phosphorylation from ATP production, which leads to heat generation^5,6^.

While a clear anatomical difference can be drawn between WAT and BAT, the cellular origin of the recently discovered beige adipose tissue (BeAT) has not been completely clarified^7,8^. Beige fat is mainly found in subcutaneous white fat depots and share many characteristics of BAT, such as enriched mitochondria, multiple lipid droplets, and expression of UCP1. Activation and recruitment of BAT and BeAT by various stimuli increases lipolysis and upregulates UCP1 causing increased whole-body energy expenditure^9–12^. Therefore, brown and beige adipocytes are promising therapeutic targets to reduce obesity, and numerous research groups are investigating the effects leading to their activation^6,13^. However, traditional methods have great difficulty in detecting brown fat activation within a heterogeneous environment. A recent publication by Alonzo *et al*. showed, for the first time, the use of two-photon excited fluorescence (TPEF) microscopy to distinguish WAT from BAT^14^. Our goal was to further investigate the potential of label-free TPEF microscopy to quantify changes in fat metabolism and activation of brown and beige adipocytes over time. One approach is the optical detection of the omnipresent metabolic co-factor nicotinamide adenine dinucleotide (NADH), which allows for non-invasive functional imaging of cellular metabolism and has been used to monitor a wide variety of cells, tissues, and organs *in vitro* as well as *in vivo*^15^. NADH plays crucial roles in both oxidative metabolism and glycolysis, and monitoring its auto-fluorescence is useful as an indicator of metabolic activity^16^. While NADH is fluorescent, its oxidized counterpart, NAD^+^, is not.

**Figure 1:**
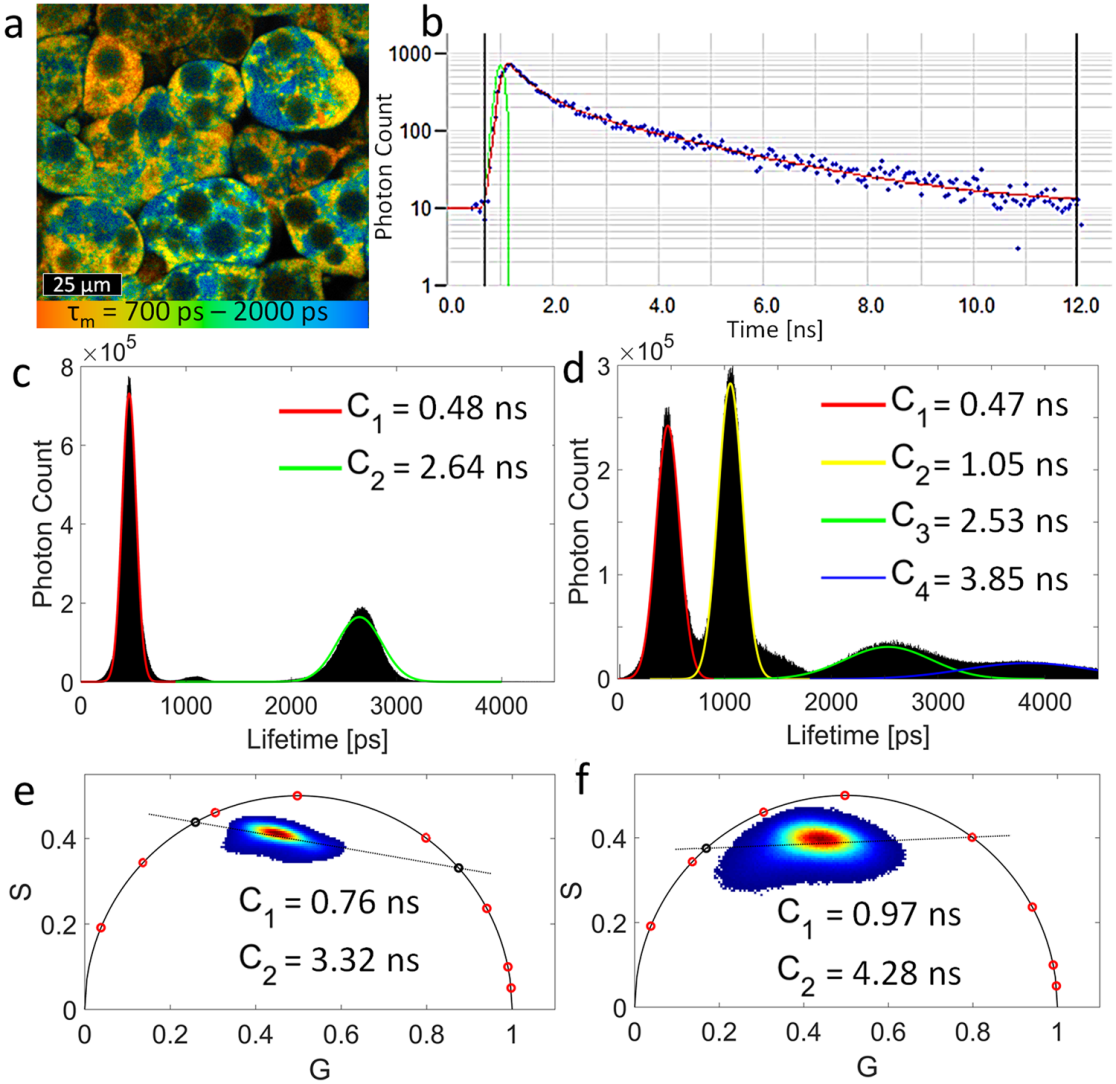
NADH fluorescence lifetime distribution of adipocytes using the phasor approach and exponential fitting of the decay curve. (**a**) FLIM image of NADH of *in vitro* 3T3-L1 adipocytes color coded for the average fluorescence lifetime. (**b**) Fluorescence decay curve of several spatially binned pixels of the FLIM image Blue: Photons collected in each time channel. Green: Instrument response function which is the pulse shape the FLIM system records for an infinitely short fluorescence lifetime. Red: Bi-exponential fit of the decay curve. NADH histogram based on the intensity and lifetime of all pixels of the FLIM image using bi-exponential fitting with a spatial binning of (**c**) 9 × 9 pixels (14 µm^2^) and (**d**) 3 × 3 pixels (1.5 µm^2^). Phasor plots of all pixels of the FLIM image showing the NADH lifetime distribution for (**e**) 9 × 9 pixels spatial binning and (**f**) 3 × 3 pixels spatial binning. The red circles on the universal circle mark 0.1 ns, 0.2 ns, 0.5 ns, 1 ns, 2 ns, 3 ns, 5 ns, and 10 ns from right to left.

Fluorescence lifetime imaging microscopy (FLIM) has been used extensively to analyze NADH which can be found as either a free molecule or bound to a variety of enzymes within cells^17^. While the fluorescence spectrum of free and bound NADH is very similar, the lifetimes of these species differ significantly. Free NADH in solution has two fluorescence lifetime components, which can be attributed to a folded and extended conformation of the molecule. In the folded conformation light absorption initiates an effective energy transfer within the molecule resulting in shorter fluorescence lifetimes compared to the extended conformation^18–22^. Additionally, parameters such as solvent medium, pH, temperature, and viscosity affect the fluorescence lifetime of NADH in solution. Binding to an enzyme prolongs the fluorescence lifetime of NADH, and the lifetime varies slightly for each of NADH’s multiple binding partners^23^. Previous reports also determined that NADH typically exists in an extended conformation when bound to a protein^18^. While traditional assessment of NADH in cell cultures and tissue only shows two fluorescence lifetimes components, one free and one bound, recent studies in cancer cells and cerebral tissue suggest that there are likely four distinct NADH species^16,24^. The cause of this are several free and individual enzyme bound species of NADH^16,24,25^. The NADH fluorescence decay can be modeled as the sum of two or more decaying exponentials, with each term representing a different conformation or enzymatic bound formulation of NADH^16^. An alternative method of analyzing lifetime data is the Fourier-based phasor approach, which calculates phasor coordinates describing the fluorescence decay (Fig. 1)^26^.

Here we present our results using FLIM to investigate heterogeneous changes in NADH activity of adipocytes. We show that different enzymatic binding states and free-to-bound ratios detected by FLIM can be used as specific biomarkers of molecular pathways associated with glycolysis or oxidative phosphorylation. To do this, NADH fluorescence lifetime data was acquired while cells were treated with a range of pharmacological agents known to affect key cellular metabolic processes. Additionally, we created a new optical ratio for enhanced quantification of metabolic activity by expanding the traditional two-lifetime model of NADH to a four-lifetime model. Mapping the four lifetime components to specific cellular components quantitatively reflects changes in mitochondrial and cytoplasmic NADH concentrations and resulted in superior metabolic assessment compared to traditional FLIM analysis. Further, it was evaluated whether changes in fluorescence lifetime correlate with gene expression of BAT-related markers such as UCP1. By understanding how fluorescence lifetime can be related to metabolic activity, this study constitutes a powerful imaging toolkit for cellular metabolism investigations and a valuable tool in obesity research.

## Results

### Spatial binning of the decay curve has a significant effect on subsequent fluorescence lifetime analysis

Recently, several articles analyzing the fluorescence lifetime of NADH were published, describing a double exponential fit and high spatial binning (>35 µm^2^), which yields two distinct fluorescence lifetimes^14,27^. The use of spatial binning increases the number of pixels and photon counts that contribute to the fluorescence decay while decreasing the effective resolution and spatial detail. However, other recent publications showed that NADH in cerebral tissue and cancer cells can have up to four distinct fluorescence lifetimes^16,24^. In contrast to studies showing only two fluorescence lifetimes for NADH, these publications made use of high resolution imaging and low spatial binning (<10 µm^2^). To determine if spatial binning affects the number of uniquely detected NADH lifetimes of adipocytes, we analyzed identical FLIM data sets using two levels of binning.

We show that reducing spatial binning from 9 × 9 pixels (14 µm^2^) to 3 × 3 pixels (1.5 µm^2^) during FLIM analysis of NADH causes an increase in the number of unique lifetime components. In the case of exponential fitting, high spatial binning (low effective spatial resolution) resulted in two fluorescence lifetimes for NADH (Fig. 1c), while low spatial binning (high effective spatial resolution) clearly resulted in four distinct lifetime components (Fig. 1d). Similarly, phasor analysis of high spatial binning images resulted in two clear lifetime contributions that are represented by two projected points on the universal semicircle (Fig. 1e). While the phasor plot generated by low spatial binning is not as straightforward to interpret, the triangular arrangement strongly suggests that more than two fluorescence lifetimes were present, even though they cannot be directly distinguished (Fig. 1f). A common method to separate components having the same phasor location but different lifetime distributions is the multi-harmonic lifetime analysis, where higher harmonics of the laser repetition rate are used^28^. For this study, this method did not result in improved separation of the phasor populations (Supplementary Figs 8 and 9 and Note 9).

### Lifetime Localization

For low spatial binning and bi-exponential fitting the NADH fluorescence lifetime distribution of the image shows four distinct fluorescence lifetimes. Even though 4 distinct fluorescence lifetimes for NADH are found, only two components are fit at each pixel. The 4 lifetimes components are only identified across different pixels and not within one fluorescence decay. The reason is that the use of a bi-exponential model to fit the fluorescence decay always returns two fluorescence lifetimes, regardless of how many lifetimes the analyzed pixel actually has. The two-lifetime model was selected as attempts to fit the data with three lifetime components did not deliver a significant improvement based on the quality of the fit (Supplementary Fig. 5 and Note 5)^29^.

**Figure 2:**
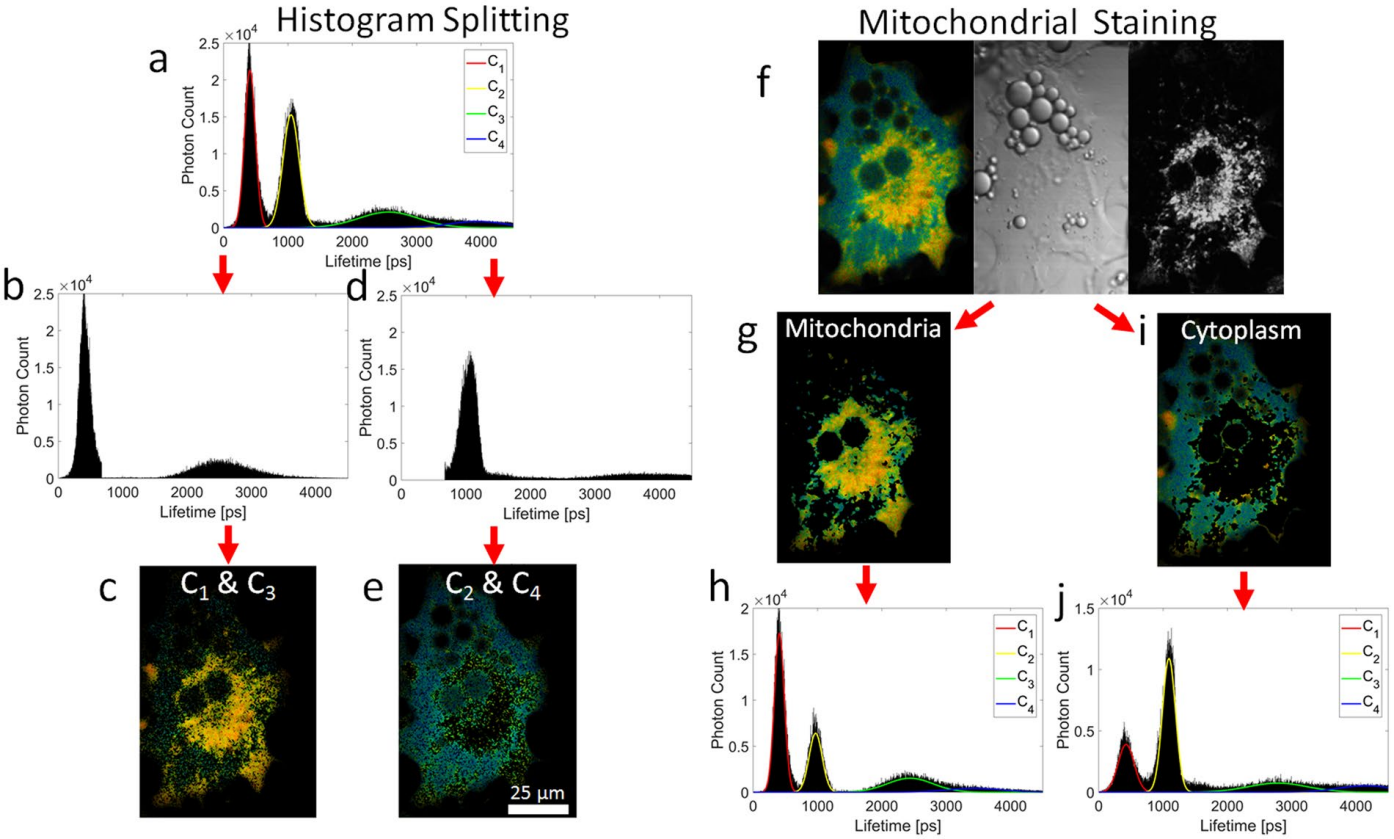
Lifetime component localization. (**a**) NADH lifetime distribution of the entire cell and Gaussian fitting of each lifetime component. (**b**,**c**) FLIM image and fluorescence lifetime distribution that only consist of lifetime pair C_1_ and C_3_. (**d**,**e**) FLIM image and lifetime distribution that only consist of lifetime pair C_2_ and C_4_. (**f**) FLIM, trans-illumination, and mitochondrial-stain image of a single 3T3-L1 fat cell. (**g**,**h**) Fluorescence lifetime distribution and FLIM image of mitochondria. (**i**,**j**) Fluorescence lifetime distribution and FLIM image of the cytoplasm.

By analyzing each pixel individually, it became clear that the lifetime components occur in pairs: the lifetime component C_1_ at 0.5 ns is predominantly accompanied by component C_3_ at 2.3 ns (Fig. 2b) and lifetime component C_2_ at 1 ns is accompanied by component C_4_ at 3.7 ns (Fig. 2d). Based on this information, two FLIM images were generated: one that shows the spatial distribution of lifetime pair C_1_, C_3_ (Fig. 2c) and another one for lifetime pair C_2_, C_4_ (Fig. 2e). As NADH fluorescence is known to arise from mitochondria, the cellular mitochondria distribution was determined using the stain tetra-methyl-rhodamine ethyl ester (TMRE) and MitoTracker Green FM. The mitochondrial stain, trans-illumination and FLIM image enabled the generation of FLIM images that belong to specific cellular regions such as mitochondria and the cytoplasm. Interestingly, these FLIM images of specific cellular regions show significant differences in fluorescence lifetime distribution. The mitochondrial lifetime distribution (Fig. 2h) and the cytoplasmic distribution (Fig. 2j) indicate that the NADH fluorescence lifetime of mitochondria favors lifetime components C_1_ and C_3_ while the cytoplasm favors components C_2_ and C_4_. Comparing the images revealed that the mitochondrial-stain derived mitochondria FLIM image (Fig. 2g) shows a high correlation (R = 0.75) with the C_1_ and C_3_ FLIM lifetime pair image (Fig. 2c) and that the cytoplasm FLIM image (Fig. 2i) has a similarly strong correlation (R = 0.67) with the C_2_ and C_4_ FLIM lifetime pair image (Fig. 2e). This supports the conclusion that lifetime components C_1_ and C_3_, are associated with mitochondria, and components C_2_ and C_4_ are observed to arise from the cytoplasm. This theory of regional differences of NADH species was further confirmed by analysis of isolated mitochondria of 3T3-L1 adipocytes which resulted in a fluorescence lifetime distribution of predominately lifetime components C_1_ and C_3_ (Supplementary Fig. 10 and Note 10).

**Figure 3:**
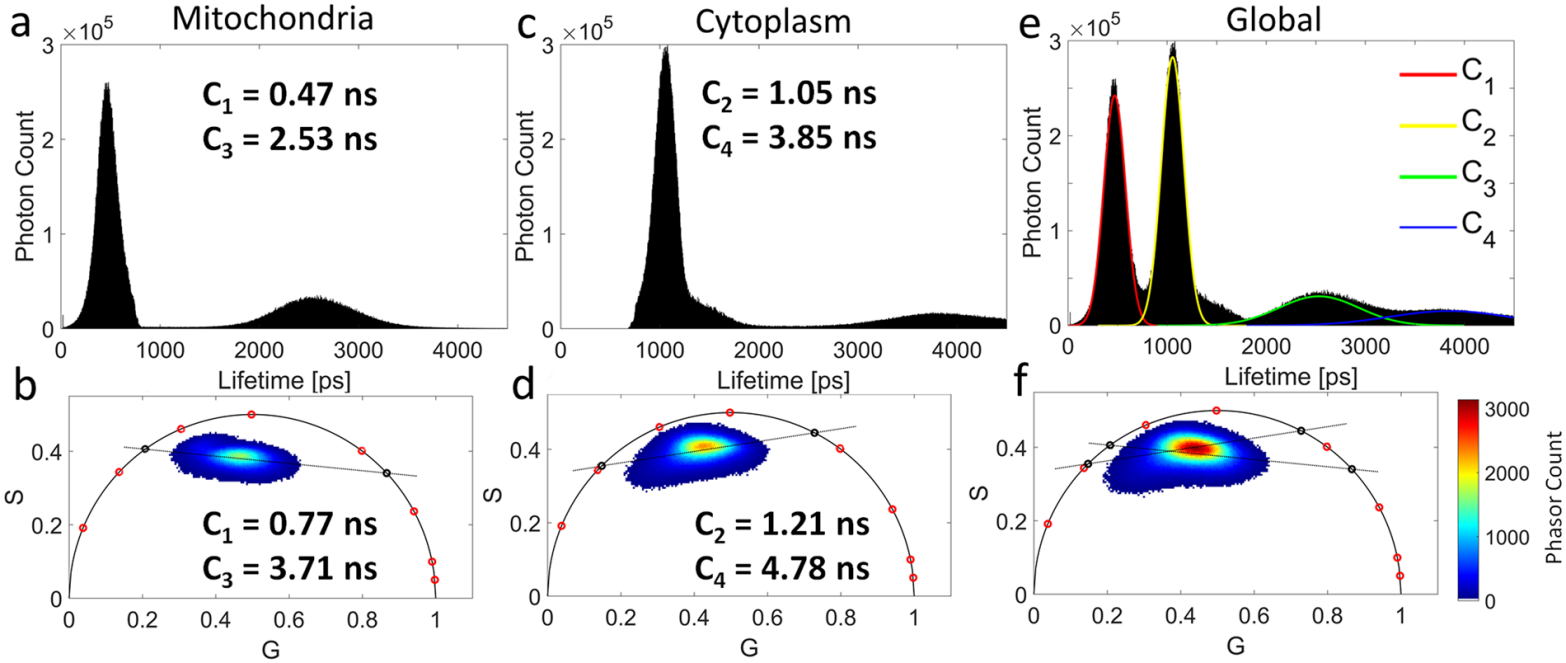
NADH lifetime distributions and corresponding phasor plots using spatial binning of 3 × 3 pixels. (**a**,**b**) mitochondrial lifetime pair C_1_ and C_3_ (**c**,**d**) cytoplasmic lifetime pair C_2_ and C_4_ (**e**,**f**) the entire FLIM Image. n = 20 FLIM images per group.

Figure 1f presented a phasor plot that arranged in a broad cluster, making it difficult to extract the correct fluorescence lifetimes. Here, we present an approach to determine the fluorescence lifetimes of broadly clustered phasor plots by combining a lifetime distribution segmentation with the phasor analysis. Therefore, a segmentation of mitochondrial and cytoplasmic NADH fluorescence is performed using the NADH lifetime distribution of lifetime pairs C_1_, C_3_ and C_2_, C_4_, respectively (Fig. 3a,c). Phasor points generated by a segmentation of mitochondrial NADH fluorescence are closely arranged along a single line (Fig. 3b) suggesting two NADH fluorescence lifetimes for this cellular compartment. The cytoplasmic phasor point distribution of NADH fluorescence (Fig. 3d) shows a slightly triangular shape. We hypothesize that the use of improved mitochondrial and cytoplasmic segmentation should also lead to a phasor point distribution along a single line, thus, representing two NADH fluorescence lifetimes for the cytoplasm. In Fig. 3, we can intuitively observe that the global phasor plot (Fig. 3f) can be described by individual fluorescence lifetimes of mitochondrial and cytoplasmic segmentation. Even though the NADH lifetimes determined by phasor analysis (Fig. 3b,d,f) are longer than the ones generated by bi-exponential fitting (Fig. 3a,c,f), the trends match well with shorter lifetime pairs associated with mitochondrial fluorescence, and longer pairs for cytoplasmic fluorescence. As the localization of each of the four lifetime components is highly specific to mitochondrial and cytoplasmic cellular features, we can define a new metabolic ratio, namely the mitochondrial-cytoplasmic-ratio (MCR), which is specific to changes in mitochondrial to cytoplasmic NADH fluorescence intensity:

1 $$MCR=\frac{Mitochondrial\,NADH\,Fluorescence}{Cytoplasmic\,NADH\,Fluorescence}=\frac{{C}_{1}+{C}_{3}}{{C}_{2}+{C}_{4}}$$

The MCR is sensitive to shifts in mitochondrial and cytoplasmic NADH concentration as well as shifts in spatial distribution of these cellular compartments. In this study, FLIM measurements were performed within minutes of the injection of pharmacological reagents. Therefore, it is likely that the MCR represents shifts in mitochondrial and cytoplasmic metabolism rather than changes in content. In the following analysis of metabolic changes, we compare the MCR to traditional optical biomarkers such as the fluorescence lifetime based free-to-bound ratio of NADH and the fluorescence intensity based redox ratio of NADH and flavin adenine dinucleotide (FAD) which can be expressed as FAD/(FAD + NADH)^30,31^.

### FLIM analysis parameters for the mitochondria stress test correlate with those determined using extracellular flux analysis

**Figure 4:**
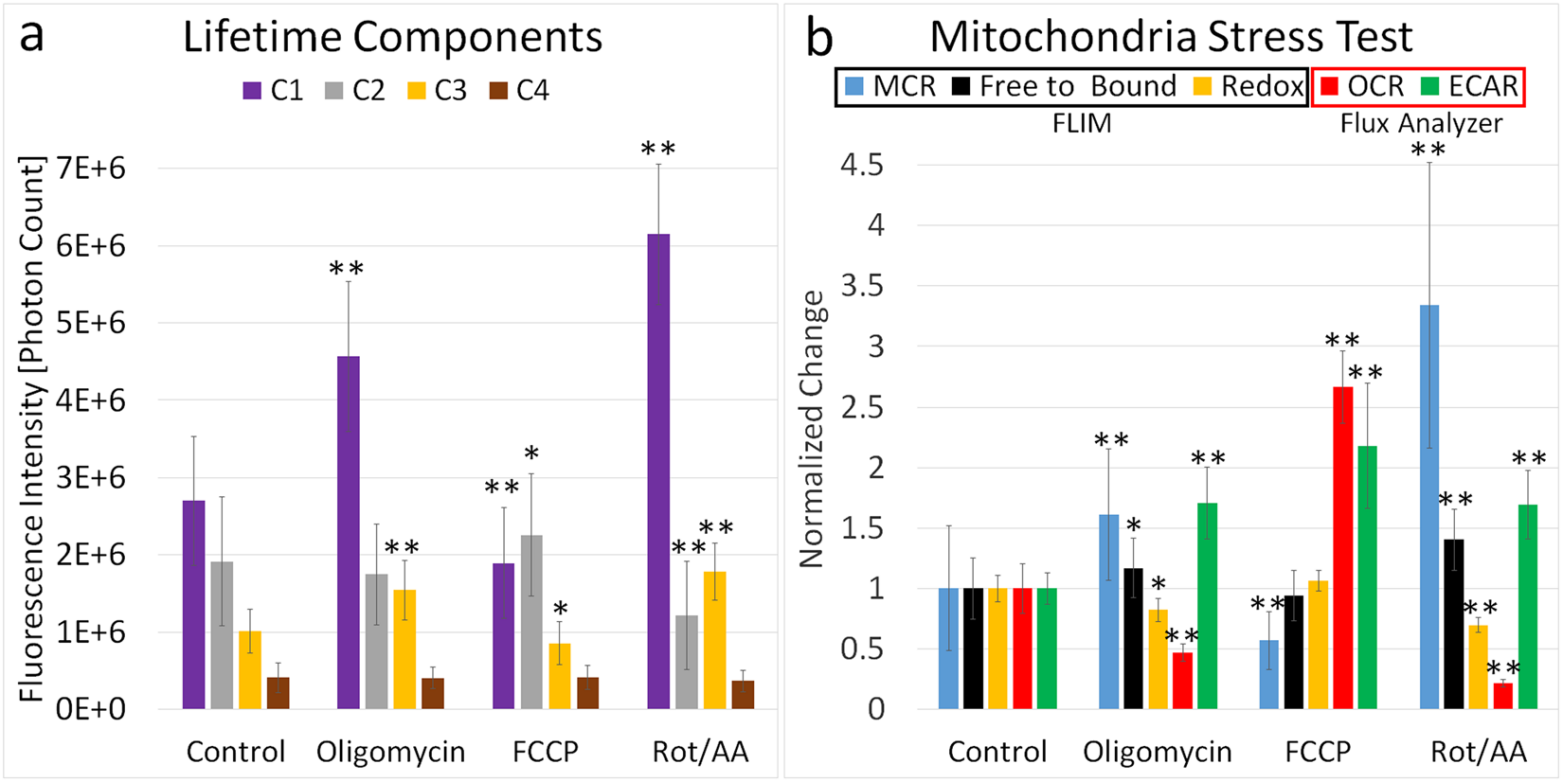
Mitochondria stress test. (**a**) Fluorescence intensity of the individual lifetime components of NADH for each step of the mitochondria stress test. (**b**) The effect of individual injections of oligomycin, FCCP, and rotenone/antimycin on the FLIM-derived MCR, optical free-to-bound ratio, and redox ratio as well as on OCR and ECAR measured by an extracellular flux analyzer. Mean ± SD of n = 40 FLIM images per group. Significant differences of treatment groups to the control by Student’s t-test with *p < 0.05, **p < 0.001.

Mitochondrial respiration and extracellular acidification were analyzed using an extracellular flux analyzer and compared to the microscopically derived optical redox ratio, free-to-bound ratio and MCR. The mitochondria stress test presented here, consisted of 3 individual injections of pharmacological reagents, the first injection being oligomycin. Oligomycin inhibits ATP synthase and prevents protons from re-entering the mitochondrial matrix, causing the inhibition of oxidative phosphorylation and consequently causing a decrease in oxygen consumption rate (OCR) along with an increase in glycolysis. As these changes drive an increase in mitochondrial NADH and decrease of FAD due to inhibition of the electron transport chain (ETC), both the optical free-to-bound ratio and MCR should increase significantly, while the redox ratio should decrease. As can be seen in Fig. 4, the optical free-to-bound ratio and MCR do indeed increase and the redox ratio decreases. Specifically, the contribution to the total fluorescence intensity of mitochondria lifetime components C_1_ and C_3_ is observed to rise, while the contribution of the cytoplasmic component C_2_ decreased. To compensate for the inhibition of the ETC, glycolytic ATP production is upregulated to sustain cellular demand, which explains the decrease of lifetime component C_2_. As expected the oligomycin injection resulted in an increased extracellular acidification rate (ECAR) determined by monitoring extracellular pH changes measured by an extracellular flux analyzer.

The second injection of the stress test is FCCP, which acts as a protonophore to translocate protons into the inner membrane of the mitochondria, accelerating respiration and thereby oxidizing NADH to non-fluorescent NAD^+^ and FADH_2_ to fluorescent FAD. The FCCP injection resulted in an increase in OCR as measured by the flux analyzer. By uncoupling oxidative phosphorylation from ATP production, FCCP causes cells to switch to glycolysis, which is marked by an increased ECAR measured by the flux analyzer. These changes cause the mitochondrial NADH pool to deplete due to higher activity of the ETC. The cytoplasmic NADH pool showed a slight increase in form of increased intensity of lifetime component C_2_, due to significantly higher glycolytic function. As anticipated, the MCR was observed to decrease, with the mitochondrial C_1_ and C_3_ fluorescence intensity declining while the C_2_ intensity increased. The redox ratio does not show significant changes compared to the baseline after FCCP injection which has been reported previously^32,33^.

The third injection of the stress test are respiratory chain inhibitors rotenone and antimycin which induce oxidative stress and maximize mitochondrial NADH by preventing its oxidation to NAD^+^, as well as prevention of oxidization of FADH_2_ to FAD. Therefore, the redox ratio decreased significantly. As expected the rotenone/antimycin injection led to a decreased OCR measured by the flux analyzer and a corresponding increase in the FLIM-derived free-to-bound ratio and MCR due to inhibition of the ETC. The injection led to maximized mitochondrial NADH which increased the C_1_ and C_3_ fluorescence intensity. As a result of the oxidative phosphorylation inhibition, glycolysis becomes upregulated in the cytoplasm, which can be seen by an increased ECAR.

### FLIM analysis parameters correlate with extracellular flux analysis for glycolytic function

**Figure 5:**
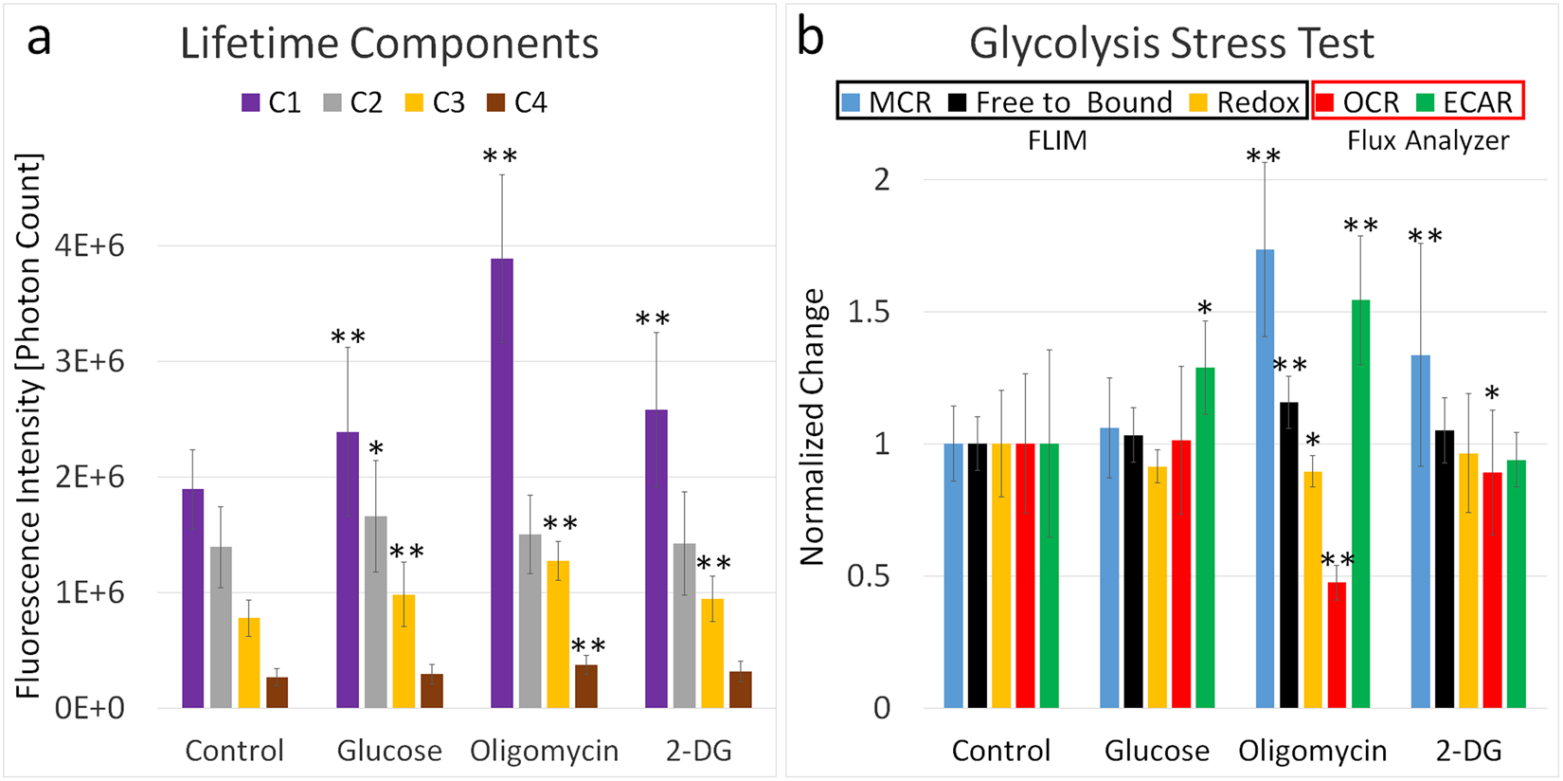
Glycolysis stress test. (**a**) Fluorescence intensity of the individual lifetime components of NADH for each step of the glycolysis stress test. (**b**) The effect of individual injections of glucose, oligomycin, and 2-DG on the FLIM-derived MCR, optical free-to-bound ratio, and redox ratio as well as on OCR and ECAR measured by an extracellular flux analyzer. Mean ± SD of n = 40 FLIM images per group. Significant differences of treatment groups to the control by Student’s t-test with *p < 0.05, **p < 0.001.

The glycolysis stress test is the standard assay for measuring glycolytic function, and for this particular stress test, cells are incubated in glucose- and pyruvate-free media. The glycolytic rate is determined by monitoring extracellular pH via changes in media lactate concentration. The glycolysis stress test presented here, consisted of 3 individual injections of pharmacological reagents. In the first step of the test, injection of glucose leads to a saturation of glucose concentration within the cell, and triggers the start of catabolism through the glycolytic pathway. This catabolic process is accompanied by a discharge of protons into the media, which was observed to cause an increase in ECAR while the OCR remained unchanged. To our surprise, the optical ratios also remained unchanged (Fig. 5). However, the fluorescence intensity of lifetime components C_1_, C_2_, and C_3_ increased significantly after the injection of glucose, suggesting higher metabolic activity. The increase of component C_2_ was expected and can be explained by reduction of NAD^+^ to NADH as glucose is broken down to pyruvate during glycolysis. The increase of mitochondrial components C_1_ and C_3_ are somewhat surprising, since no increase in OCR occurred. This could be explained by increased activity in the tricarboxylic acid cycle induced by pyruvate shuttled into the mitochondria^34^.

In the second step, injection of oligomycin reveals the maximum glycolytic capacity by inhibition of mitochondrial energy production, causing a shift in the ATP production to glycolysis. The ECAR measured by the flux analyzer, and the FLIM-derived MCR, C_1_, C_3_ and C_4_ lifetime component fluorescence were observed to increase significantly after the injection of oligomycin, while the OCR decreased significantly. As expected, the oligomycin injection of the glycolysis stress test (Fig. 5) has an effect on the cells very similar to the mitochondria stress test (Fig. 4). Given both the microscopy- and flux analyzer-derived data, it is very likely that the changes of lifetime components C_1_ and C_3_ following oligomycin injection into the media are caused by inhibition of the ETC while fluorescence of component C_4_ increased due to higher glycolytic activity.

In the final stress test step, 2-DG is injected to inhibit glycolysis by binding to the first enzyme of the glycolytic pathway showing the non-glycolytic acidification of cells, leading to an observed decrease in OCR without a resulting change in ECAR as determined by the flux analyzer. Unchanged ECAR after 2-DG injection was anticipated since control cells have a very low glycolytic rate due to the use of glucose- and pyruvate-free cell culture media. The FLIM-derived MCR, component C_1_ and C_3_ all increased compared to the control. These change s, which correspond to a decreased OCR measured by the flux analyzer, can be explained by reduced activity of the ETC.

### Adipocytes show increased metabolism due to browning reagents

**Figure 6:**
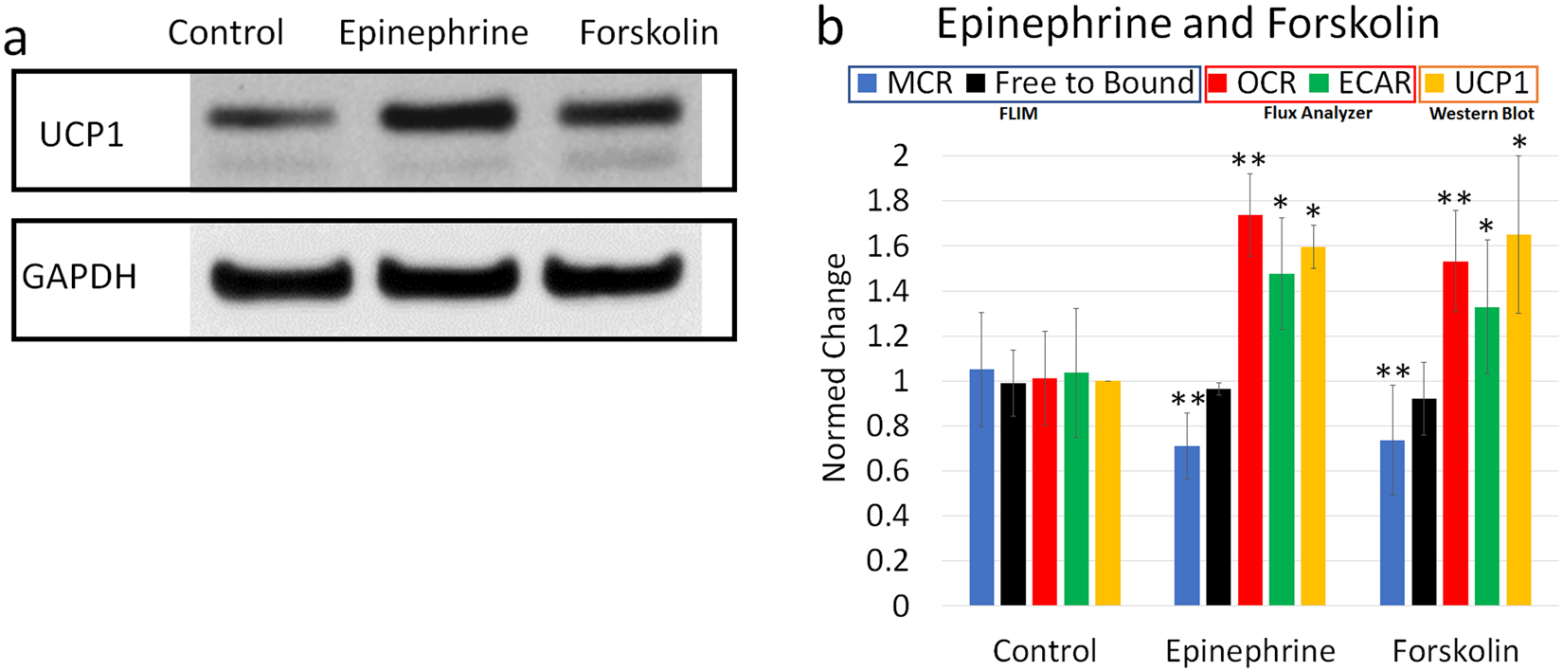
Murine white inguinal fat after 1-hour treatment with epinephrine and forskolin compared to untreated cells. (**a**) Expression of UCP1 and housekeeping gene GAPDH. UCP1 expression is normalized to the expression of GAPDH. Full length western blots are shown in Supplementary Fig. 6. (**b**) Effect on the FLIM-derived free-to-bound ratio and MCR, OCR and ECAR measured by the extracellular flux analyzer, and the gene expression of browning marker UCP1 analyzed by western blotting. Mean ± SD of n = 20 FLIM images per group. Significant differences of treatment groups to the control by Student’s t-test with *p < 0.05, **p < 0.001.

Browning of white adipocytes has been described as an increase in UCP1 expression, resulting in thermogenic, fat-burning properties^10^. Though they initially show low levels of UCP1 expression, exposure of white adipocytes to mild cold or pharmacological reagents can activate a thermogenic pathway that induces the expression of UCP1, and lipolysis. To examine this effect, epinephrine and forskolin, which both promote lipolysis and are known to induce browning, were added to inguinal adipocytes in culture for 1 hour. Western blots (Fig. 6a) show increased levels of UCP1 protein for treated cells compared to untreated cells. UCP1 expression was normalized to the expression of reference gene GAPDH which remained constant under investigation. Like the pharmacological reagent FCCP, UCP1 acts as a protonophore to facilitate proton translocation across the inner membrane of the mitochondria. Figure 6 shows that like FCCP (Fig. 4), both epinephrine and forskolin cause a significant increase in OCR and ECAR. While the optical free-to-bound ratio had no significant change, it is interesting to note that the new MCR metric decreased due to small but significant changes in lifetime components C_1_ and C_2_ after treatment. Furthermore, it is shown that the MCR is inversely proportional to the OCR, further supporting the idea that this metric is sensitive to metabolic changes of the ETC (Figs 4, 5 and 6).

## Discussion

This study shows, for the first time, that four fluorescence lifetime components exist for NADH in adipocytes, and that lifetime pairs can be localized to distinct cellular compartments. The fluorescence lifetime component C_1_ (0.5 ns) and C_3_ (2.3 ns) are predominantly found in mitochondria, while the lifetime components C_2_ (1 ns) and C_4_ (3.7 ns) predominantly arise from the cytoplasm. This is in contrast to the traditional two lifetime model for NADH, where the short fluorescence lifetime τ_1_ (0.5 ns) represents the cytoplasm and the long fluorescence lifetime τ_2_ (2.5 ns) is attributed to the mitochondria^25^. In the two-lifetime model, it is believed that the cytoplasm, where glycolysis occurs, is associated with free NADH and mitochondria, where oxidative phosphorylation takes place, are associated with bound NADH. Hence, the free-to-bound ratio has traditionally been thought of as a glycolysis/oxidative phosphorylation ratio. In this traditional model, NADH within the mitochondria binds to several enzymes and proteins such as lactate dehydrogenase (LDH) and malate dehydrogenase (MDH) and a change of the free-to-bound ratio can therefore be seen as a change in enzyme binding.

**Figure 7:**
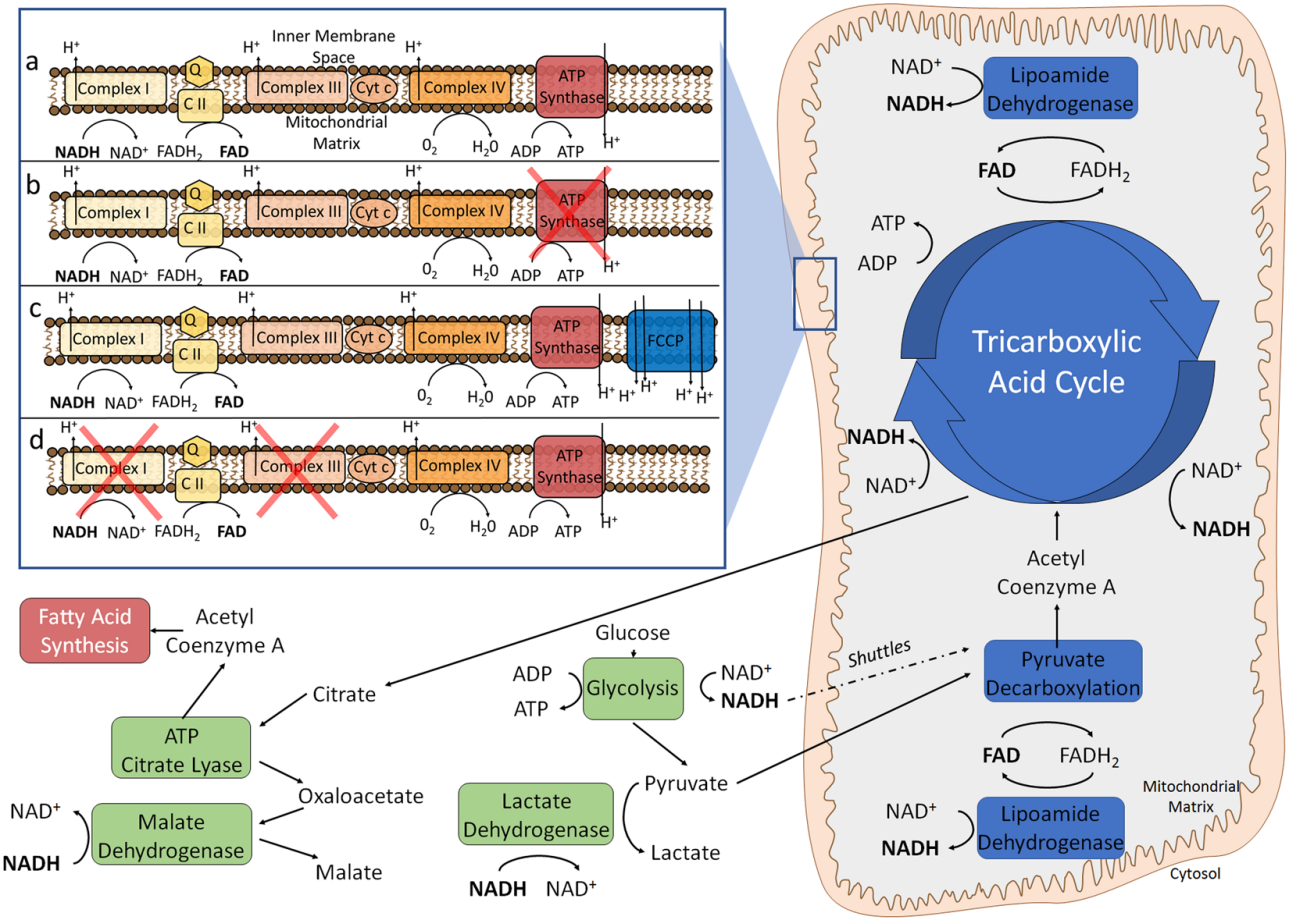
Simplified schematic of glycolysis and oxidative metabolism that highlights the roles of the intrinsic fluorophores NADH and FAD and shows the ETC and the effect of pharmacological reagents. (**a**) Baseline (**b**) Oligomycin inhibits the ATP synthase (**c**) FCCP acts as a protonophore (**d**) Rotenone and Antimycin inhibit complex I and III.

This traditional explanation, which relies on the theory that bound NADH is mainly found in mitochondria, is however limited in its application since NADH also binds to cytosolic enzymes such as glyceraldehyde phosphate dehydrogenase (GAPDH) and LDH (Fig. 7)^27,35^. Additionally, measurements with NADH in solution show that it has two intrinsic fluorescence lifetimes which most likely represent different conformations of free NADH, possibly folded (0.4 ns) and extended (0.9 ns)^15,23^. Interestingly, the fluorescence lifetimes of free NADH in solution reasonably concur with lifetime components C_1_ (0.5 ns) and C_2_ (1 ns) of NADH in adipocytes. Mixing pure NADH in solution with LDH, an enzyme which is found in mitochondria as well as the cytoplasm, introduces a third fluorescence lifetime component to the solution (Supplementary Fig. 2 and Note 3). These findings, lead to the assertion that both cellular features must each have free and bound NADH fluorescence lifetimes. The difference between free and bound NADH lifetimes of mitochondria (0.5 ns and 2.3 ns) and the cytoplasm (1 ns and 3.7 ns) may be attributable to different micro-environmental conditions such as pH, viscosity, and/or osmotic concentration. We conclude that a 4-lifetime component model is entirely appropriate, with the MCR as a useful new tool to quantify mitochondrial and cytoplasmic NADH changes.

**Figure 8:**
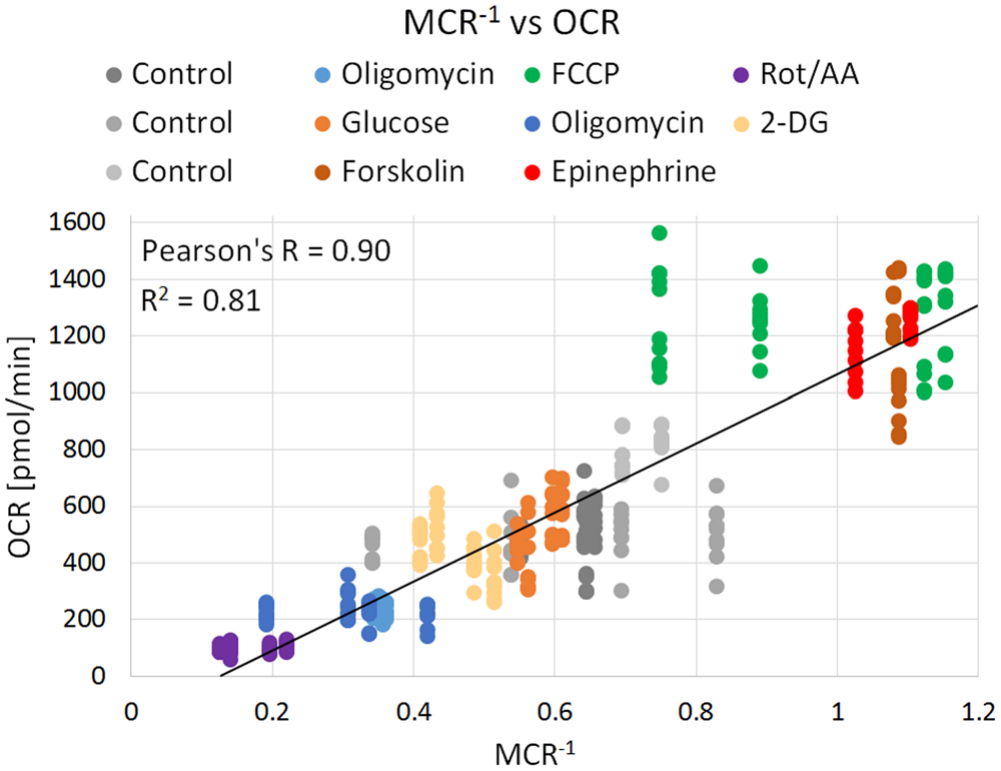
Scatter plot of OCR vs MCR^−1^ for all experimental conditions at 20,000 cells per well. A linear regression was fitted through the OCR and MCR^−1^ data of 3T3-L1 and primary inguinal adipocytes for controls and after injection of various pharmacological reagents. The R^2^ value which shows how close the data are to the fitted regression line was calculated. The Pearson’s correlation shows the measure of strength and direction of association that exists between the OCR and MCR^−1^.

The mitochondria stress test (Fig. 4) showed that oligomycin and rotenone/antimycin injections inhibit the ETC, which maximizes NADH within the mitochondria. This leads to an increase in fluorescence intensity contribution for mitochondria-associated lifetime components C_1_ and C_3_ and a decrease for cytoplasmic-associated lifetime component C_2_. The increase of absolute fluorescence intensity of C_1_ and C_3_ was expected since Vergen *et al*. showed that inhibition of the ETC leads to an increase in mitochondrial NADH and quantum yield^24^. FCCP acts to increase mitochondrial respiration by uncoupling ATP phosphorylation from the ETC which depletes the mitochondrial NADH pool. While this metabolic alteration does not affect the traditional free-to-bound and redox ratios, a significant change can be detected with the MCR metric (Fig. 4). This highlights the strength of the 4-lifetime analysis of NADH, as the decreased MCR arises from subtle changes of lifetime components C_1_ (0.5 ns) and C_2_ (1.0 ns) which are undetectable in the traditional two-lifetime analysis. The results of Figs 4, 5 and 6 suggest that while the free-to-bound ratio, the redox ratio and the MCR have reproducible results, the MCR has a greater magnitude of response to changes of the ETC. Furthermore, it is shown that MCR is inversely proportional to the OCR, supporting the idea that this metric is sensitive to metabolic changes of the ETC (Fig. 8). Even though a strong correlation exists between MCR^−1^ and OCR the control data appears at different points on the plot (Fig. 8). This can be explained by different cell culture conditions such as type of media and cells. In future studies, we will look further into the predictive relationship of MCR^−1^ and OCR. Compared to the free-to-bound and redox ratio, the MCR represents dynamic metabolic changes with high sensitivity and enables FLIM as a visual alternative to the flux analyzer, especially for applications in heterogeneous cell cultures and even tissue.

The level of visual image detail could be further improved using higher numerical aperture objective lenses and abstaining from spatial binning in the subsequent analysis. This combination would increase the spatial sampling resolution of the FLIM analysis significantly. It is worth noting that doing so would also require collection of far more photons to achieve the signal-to-noise ratio necessary to facilitate accurate fitting of the fluorescence decay traces. Even though higher photon yield could be achieved by longer imaging durations or higher excitation intensity, it might also lead to photobleaching, which could distort lifetime measurements and lead to significant error. Cellular photo damage threshold calculations as well as photobleaching experiments showed that the used laser parameters did not induce immediate or long-term cell damage (Supplementary Figs 1 and 7, Table 1 and Note 1,2,8). Further enhancement for low magnifications could be achieved by adding additional exponential components during the fitting procedure as shown by Yaseen *et al*., who used 2 constrained and 2 free lifetimes resulting in a quadruple-exponential fit^36^. However, a combination of segmentation and phasor analysis may ultimately be a more powerful approach than exponential fitting, especially in heterogenous cellular or tissue environments (e.g. a tumor) where simple model-based fitting approaches can fall short.

The glycolysis stress test (Fig. 5) showed that changes that act to solely increase glycolytic activity, but that do not impact the ETC such as a glucose injection, only introduced insignificant changes by either the free-to-bound ratio, redox ratio or the MCR metric. While ECAR increased after glucose injection, the rest of the metabolic identifiers such as OCR, free-to-bound ratio and MCR remained unchanged. ECAR measures glycolytic acidification of the assay media by conversion of glucose to lactate^-^ + H^+^ and the export of products into the assay medium. Glucose catabolism to pyruvate would suggest an initial increase of cytoplasmic NADH through reduction of NAD^+^, which indeed can be seen by an increase in cytoplasmic fluorescence intensity of lifetime component C_2_ (Fig. 7). Nevertheless, the MCR did not change due to an equal fluorescence intensity increase of mitochondrial NADH. A possible explanation for this relatively small increase of NADH concentration within the cytoplasm could be the conversion of pyruvate to lactate, which involves the oxidation of NADH back to NAD^+^ (Fig. 7)^34^.

The possibility of browning of white fat is a desirable prospect for the treatment of obesity and related disorders. Therefore, white inguinal adipocytes were treated with epinephrine and forskolin which are known to activate browning. Treated adipocytes showed a similar metabolic reaction (Fig. 6) as cells treated with the uncoupling agent FCCP (Fig. 4), which can be explained by upregulation of UCP1 as shown by gene expression. UCP1, which is similar to FCCP, acts as a protonophore to facilitate proton translocation across the inner membrane of the mitochondria, which acts to downregulate aerobic ATP synthesis and up-regulate oxygen consumption^37^. Model calculations revealed consistent metabolic flux changes for both FCCP- and UCP1-mediated uncoupling, suggesting a common mechanism^37^. UCP1 expression and FCCP treatment increased glucose uptake and a substantial output of lactate as seen by an increased ECAR as well as increased mitochondrial respiration indicated by an increased OCR. These signs of increased metabolic activity which are signs of browning were also detected by the new FLIM derived MCR (Fig. 6).

This study highlights the influence spatial binning and lifetime mapping have on exponential fitting and phasor analysis of the fluorescence lifetime decay. Despite the generation of longer fluorescence lifetimes, the phasor plots of Figs 1 and 3 agree with the bi-exponential fit and support the findings of this study. Future investigations will further explore and combine both analysis modalities as we see considerable potential to further improve FLIM analysis. While spatial binning does improve photon numbers and thus the precision of a given fit, spatial binning can also average out spatial heterogeneity in the fluorescence lifetime decay traces, which would therefore result in a loss of information. If a sample exhibits a high spatial density of different fluorescence lifetimes, spatial binning of fluorescence decays will lead to an averaging out of unique lifetime components. Therefore, there must be a balance between spatial binning size to preserve spatial information and photon counts to achieve good parameter estimation, in either temporal or phasor analysis. We showed that the number of NADH lifetime components varies from two for high spatial binning (3.7 × 3.7 µm) to four for low spatial binning (1.2 × 1.2 µm). In this case, high spatial binning resulted in a loss of detail and lifetime information due to averaging (for exponential and phasor analysis) and therefore caused the observed collapse of four lifetime components to only two lifetime components. The conclusion is that subcellular structures smaller than 3.7 µm are related to the increase in number of lifetime components. The localization of individual lifetime components revealed that there is a good correlation between mitochondria and components C_1_, C_3_ and the cytoplasm and components C_2_, C_4_ (Fig. 2). Analysis of mitochondria within adipocytes using mitochondrial stains indicated tubular shapes with diameters of around 1 µm and lengths of up to 6 µm^38^. These proportions fit the binning size requirement (<3.7 µm) and could explain the impact of spatial binning on lifetime analysis (Supplementary Figs 3, 4 and Note 4).

Although the fluorescence lifetime components yield a good correlation with distinct cellular compartments, there are several causes that impair this correlation. For instance, image analysis of mitochondria-labeled cells revealed an average displacement of up to 5 µm during the typical FLIM image acquisition time of 60 s (Supplementary Figs 3, 4 and Note 4). This amount of movement leads to mitochondria entering and exiting the focal plane during the acquisition time, causing blurring and impacting the fluorescence lifetime analysis as well as a reduced correlation between FLIM images and fluorescence images of mitochondria (Fig. 2). The double-exponential model used in this work is not suited for areas of the cell that display a mixture of cytoplasmic and mitochondrial fluorescence, which can lead to suboptimal fitting of the fluorescence lifetimes. Any inaccuracy induced by mitochondrial movement acts to broaden the lifetime distribution peaks of the individual lifetime components, and makes it difficult to easily separate each one: this ultimately is the reason why the cytoplasmic phasor plot has a triangular shape (Fig. 3d). One way to reduce mitochondrial motion blur without stopping mitochondrial movement would be a reduction of the image acquisition time. It is worth noting, that reducing image acquisition times currently carries its own set of problems, as an increase in laser intensity could lead to cell damage while fewer acquisition scans would lead to suboptimal photon statistics for analysis.

Finally, the possibility that NADPH autofluorescence contributed to the results can safely be neglected, since previous studies have shown that its intracellular concentration is 4 to 40 times lower than NADH, which also has a greater quantum yield^17,31,39^. Additionally, the type I collagen coating of the glass bottom dishes, could theoretically interfere with the results of NADH fluorescence^40,41^. However, a measurement of collagen coated dishes did not show any measurable fluorescence. FAD could contribute to the measured fluorescence signal at 755 nm but its emission peak at 525 nm makes it an unlikely contributor to the NADH channel used in the FLIM system. A locally adaptive threshold (Matlab function: adaptthresh) was used to exclude regions of low fluorescence intensity such as lipid droplets and background. Lipid droplets were excluded since they are known to have long mono-exponential fluorescence lifetime decays (6.5–7,8 ns) which would interfere with the NADH fluorescence lifetime analysis^14,42^. Another lipid-based fluorophore that could interfere with the analysis is lipofuscin which excitation ranges from 360 to 660 nm with a fluorescence lifetime of about 3.2 ns^43,44^. Stimulated Raman Scattering (SRS) microscopy which is highly sensitive in the detection of lipids showed that these components didn’t affect the NADH lifetime analysis (Supplementary Fig. 13 and Note 12). Great care has been taken to achieve cell cultures of the same confluence for each experiment, since it was shown that cells of higher confluence exhibit an overall decrease in NADH lifetime and an increase in free-to-bound ratio due to lower anabolic demands of the cells^4^. Additionally, all experiments were done under the same conditions using the same protocols, since slight changes can lead to significant differences in the metabolic state of a cell. To confirm that fluorescence lifetime changes were based on cellular metabolism rather than changes in pH induced by the injection of pharmacological reagents; pH-meter measurements showed that none of the reagents resulted in a significant change of the pH-value of the media (Supplementary Table 2 and Note 6).

In summary, we have shown, for the first time, that four fluorescence lifetime components exist for NADH in adipocytes and that pairs of these lifetimes can be localized to distinct cellular compartments. This was accomplished by using high numerical aperture imaging and low spatial binning for the fluorescence lifetime analysis. It was shown that changes of the ETC and therefore of oxidative phosphorylation significantly altered the ratio of the four fluorescence lifetime components. Furthermore, we defined a new optical ratio named mitochondrial-cytoplasmic-ratio (MCR) that accurately reflects shifts in mitochondrial and cytoplasmic NADH concentration. We found that the MCR has an increased response and dynamic range to metabolic changes compared to traditional optical assessments such as the frequently used optical free-to-bound ratio. Our results show that non-invasive detection of dynamic metabolic changes and browning of live adipocytes are enabled by label-free monitoring of the fluorescence lifetime analysis of NADH. It should be noted that while this study focused on quantification of metabolic activity for adipocytes, that this approach is widely applicable and constitutes a powerful tool for studies where monitoring cellular metabolism is of key interest.

## Materials and Methods

### Cells

Mouse embryo 3T3-L1 preadipocytes were maintained in Dulbecco’s modified Eagle medium (DMEM) supplemented with 10% bovine calf serum and penicillin-streptomycin. To induce differentiation into mature adipocytes, confluent cells were cultured in DMEM containing 10% fetal bovine serum (FBS), 5 μM dexamethasone, 0.5 μg/mL insulin, and 0.5 mM isobutylmethylxanthine for 48 h to induce adipogenesis. Cells were then refed every other day with DMEM containing FBS and 0.5 μg/mL insulin for 7 days^45^.

For primary murine preadipocytes, young mice were euthanized and inguinal adipose pads were dissected, minced, and digested with collagenase at 1 mg/mL in HEPES-buffered solution at 37 °C under agitation for 45 minutes. After filtration, the cell suspension was centrifuged for 10 minutes at 100 G and the pellet containing the stromal vascular fraction was washed with DMEM media. Cells were plated in DMEM media supplemented with 10% FBS and penicillin-streptomycin. When cells reached confluence, adipocyte differentiation was initiated by addition of 5 µM dexamethasone, 0.5 µg/mL insulin, 0.5 mM isobutylmethylxanthine, and rosiglitazone 1 µM for 48 hrs. Then, cells were refed every other day with DMEM containing FBS and 0.5 µg/mL insulin. For FLIM experiments, cells were seeded into coated 96 well glass bottom culture plates. The cells were grown to confluency, and differentiated as described above. On day 7 of differentiation, medium was refreshed 8 to 12 hours before the mature adipocytes were ultimately imaged. Animal care and experiment protocols relevant to tissue dissection of euthanized mice were approved by the Institutional Animal Care and Use Committee of Massachusetts General Hospital, in accordance with NIH guidelines. Mitochondria were isolated using a Dounce homogenization procedure in conjunction with the mitochondria isolation kit for cultured cells (89874, ThermoFisher) as per the manufacturer’s instructions.

### Western Blot

Western blot analysis was used to measure the expression of traditional markers of thermogenesis such as UCP1. After treatment with forskolin and epinephrine, adipocytes were washed with phosphate-buffered saline (PBS) and lysed using mammalian protein extraction reagent containing a protease inhibitor cocktail. Protein concentration was measured using a Coomassie Plus assay kit. 100 µg of each supernatant sample of protein was separated by electrophoresis and transferred to a 0.2 µm trans-blot transfer medium nitrocellulose. Following transfer, membranes were blocked with 5% skim milk dissolved in tris-buffered saline (TBS) at room temperature for 1 h. Then, membranes were incubated overnight with primary UCP1 and GAPDH antibodies at 4 °C. Blots were washed 3 times with TBS and then incubated with horseradish peroxidase-conjugated secondary antibodies for 1 h at room temperature. Blots were washed 3 times with TBS and then treated with ECL Plus western blotting substrate. Finally, the blots were exposed to X-ray films and developed. Expression levels were quantified and normalized to GAPDH. Changes with respect to the control expression were computed and the normalized fold change in expression was compared to the change of fluorescence lifetime.

### Cellular Respiration and Extra Cellular Acidification

Oxygen consumption rate and extracellular acidification rate were determined in murine adipocytes, using a XF24 analyzer (Seahorse Bioscience). At day 6 of differentiation, cells were plated into V7-PS plates coated with collagen at 20,000 cells per well. On day 7 of differentiation and 1 hour before the OCR and ECAR experiments, cells were kept at 37 °C and not treated, treated with forskolin (5 µM), or treated with epinephrine (0.5 µM). Growth medium was replaced before treatment for both control and experimental groups. The mitochondria stress test measurement protocol to determine mitochondrial respiration consisted of injections of: (1) oligomycin (1 µM) to inhibit mitochondrial ATP production and probe uncoupled respiration; (2) carbonyl cyanide-4-(trifluoromethoxy)-phenylhydrazone (FCCP, 1 µM) to induce maximal mitochondrial respiration; and (3) rotenone/antimycin (0.5 µM) to inhibit mitochondrial respiration. After the injections, three mixing and measurement cycles of 3 minutes each were performed. The same mixing and measurement protocol was used for the glycolysis stress test to measure glycolytic function. Injections consisted of glucose, oligomycin and 2-deoxy-D-glucose (2-DG). Glucose (10 mM) is taken up by the cells and represents the rate of glycolysis under basal conditions, oligomycin (1 µM) shifts the energy production to glycolysis and reveals the maximum glycolytic capacity of the cell, and 2-DG (50 mM) inhibits glycolysis and decreases ECAR.

### Microscopy

Fluorescence intensity and lifetime imaging of two-photon excited NADH were performed on a confocal microscope (FV1000, Olympus) equipped with a tunable (680–1300 nm) ultrafast laser system (InSight DeepSee; Spectra-Physics) with a 60x water objective (NA 1.2) (UPLSAPO 60xW, Olympus) using a 2x digital zoom. The intrinsic fluorescence associated with NADH and FAD was generated at excitation wavelengths of 755 nm and 860 nm, respectively, with laser powers maintained below 15 mW at the microscope objective for all imaging experiments. NADH and FAD emissions were first separated using a 480 nm dichroic mirror. For NADH detection, a 475 ± 30 nm band-pass filter (HQ475/60 M, Chroma) was placed downstream of the dichroic mirror’s reflected optical path, such that the effective detection ranged from 445 to 480 nm. For FAD detection, a 525 ± 25 nm band-pass filter (HQ525/50 M, Chroma) was placed downstream of the dichroic mirror’s transmitted optical path. Emission events were registered by external photomultiplier tubes (PMTs; H7422p-40 for NADH detection and H7422p-50 for FAD detection, Hamamatsu) attached to a commercial time-correlated single photon counting electronics module (SPC-150, Becker & Hickl GmbH). SRS microscopy imaging was obtained by combining a 1040 nm and a 803 nm beam using a dual output femtosecond pulsed laser system (InSight DeepSee; Spectra-Physics) on the same microscope as described above. The SRS detector was placed downstream of the sample in the trans-direction. The 1040 nm beam was modulated at 20 MHz using an electro-optic modulator (ThorLabs EO-AM-R-20-C2, Newton, NJ, USA), and the signal was detected using a photodiode coupled to a lock-in amplifier (APE Lock-In Amplifier, Berlin, Germany). The adipocytes were imaged using continuous scanning with a pixel dwell time of 2 µs over a total acquisition time of 60 to 90 s to collect sufficient photon counts per pixel. For each field of view, images were acquired at a resolution of 256 × 256 pixels (105 × 105 µm). The scan of a single frame takes 375 ms including delays introduced by sawtooth scanning and flyback times of the galvanometer scanners. The PMT gain and laser power were kept constant and were measured for each image. Redox images were generated by computing pixel-wise ratios of FAD/(FAD + NADH). Cell cultures were maintained at 37 °C within a humidified 5% CO_2_ or ambient air environment using a microscope-compatible micro-incubator system (INUBTF-WSKM-F1, Tokai Hit). For confocal imaging, adipocytes were stained with Tetramethylrhodamine ethyl ester (TMRE) (Life Technologies, Molecular Probes T669) and MitoTracker Green FM (M7514, ThermoFisher) as per the manufacturer’s instructions. Fluorescence images of the mitochondria stains were performed on living cells using an inverted confocal microscope (FV1000, Olympus). Correlations between cytoplasmic and mitochondrial compartment images determined with FLIM, and mitochondrial stain images were calculated using the CORR2 function in MATLAB which implements the Pearson correlation to 2-D arrays^46^. After imaging, culture dishes with inguinal adipocytes were transferred into an incubator at 37 °C and experimental groups were treated with forskolin (5 µM) or epinephrine (0.5 µM). After 1 hour, cell cultures were imaged again to capture metabolic changes. For 3T3-L1 preadipocytes FLIM data was compared to OCR and ECAR data after applying mitochondria and glycolysis stress tests. Cell culture dishes that were imaged were treated with the same stress test protocol as cell cultures that were analyzed by the extracellular flux analyzer.

### Statistical Analyses of Fluorescence Lifetime Parameters

The fluorescence lifetime decay was spatially binned over the pixel of interest and its nearest neighboring pixels, which was then individually fitted with a double-exponential model. Spatial binning of the fluorescence decay defines how many immediate adjacent pixels are combined before the lifetime is calculated. This results in an increased accuracy of fluorescence lifetime decay times at the cost of decreased spatial resolution. Fluorescence lifetime analysis was performed using commercially available software (SPCImage, v5.7, Becker & Hickl GmbH) in combination with custom designed software developed in Matlab, where the deconvolved fluorescence intensity decay per pixel, f_2P_, is generally fit as:

2 $${f}_{2P}={\sum }_{i}{\alpha }_{i}{e}^{(\frac{-t}{{\tau }_{i}})}$$

where α_i_ and τ_i_ are the relative amplitude and lifetime of the i^th^ fluorescence component, respectively. The relative concentration of NADH associated with a given lifetime is directly related to the fluorescence decay coefficients α_i_ for a given pixel^20^. We assume that NADH is the only fluorophore contributing to the signal, where NADPH fluorescence can safely be neglected, as explained earlier^47^. Relative fluorescence contributions were calculated for each FLIM image to produce the average value and standard deviations of specific parameters such as lifetime components, corresponding coefficients and their ratios. To reduce background noise and disturbing factors, only pixels with a total photon count higher than 100 and χ^2^ values between 0.9–1.3 were used. Fluorescence lifetime histograms based on intensity were fit with a series of Gaussian functions suitable to the number of peaks required to acquire a satisfying fit:

3 $${C}_{i}={a}_{i}{e}^{-\frac{{(x-{b}_{i})}^{2}}{2{c}_{i}^{2}}}$$

where *a* is the height of the *i*^*th*^ lifetime component peak, *b* is the position of the center of the peak and *c* controls the width. The area of each individual Gaussian function was computed and were used to determine individual lifetime components and ratios, and the peak positions were utilized to define the mean lifetime for each of the NADH pools. Pearson correlation and linear regression of the OCR vs MCR^−1^ data were calculated using the cor.test and lm function in R, respectively.

### Phasor Analysis

The phasor plot is a graphical and fit-free representation of raw FLIM data. Phasor plots were generated by a custom designed software developed in Matlab based on the source code of Lanker *et al*.^48^. The intensity *I(t)* of a time-resolved fluorescence decay recorded at each pixel location can be plotted as a single point in the phasor plot by applying the sine and cosine transforms to the measured decay data. This is equivalent to the real and imaginary components of the Fourier transform of the decay data:

4 $$g(\omega )=\frac{{\int }_{0}^{\infty }I(t)\cos (\omega t)}{{\int }_{0}^{\infty }I(t)}$$

5 $$\,s(\omega )=\frac{{\int }_{0}^{\infty }I(t)\sin (\omega t)}{{\int }_{0}^{\infty }I(t)}$$

where ω is the laser repetition angular frequency, defined as ω = 2π*f*, with *f* = 80 MHz for the ultrafast laser source used in all imaging experiments. The universal semicircle is a lifetime ruler in the phasor plot, where the lifetimes increase counterclockwise from right (1,0) (lifetimes approaching zero) to left (0,0) (lifetimes approaching infinity). A phasor population falling on the semicircle indicates a mono-exponential decay, while multi-exponential decays fill the area inside the semicircle as linear combinations of their contributing mono-exponential components. Principal component analysis was used to create fitted linear functions on the universal circle to determine fluorescence lifetimes. As shown in Fig. 1, the phasor plot of NADH, composed of two lifetimes, lies on a fitted line connecting it to the semicircle, where the two intersecting points between the line and the semicircle represent each individual lifetime^26,49^. Fluorescein fluorescence was used as a reference to correct for the instrumentation response function^50^.

### Data Availability

The datasets generated during and/or analyzed during the current study are available from the corresponding author on reasonable request.

## Electronic supplementary material

Enhanced quantification of metabolic activity for individual adipocytes by label-free FLIM
