# Supplementary material for: Enhanced quantification of metabolic activity for individual adipocytes by label-free FLIM
Source: Sci Rep. 2018 Jun 8;8:8757. doi: 10.1038/s41598-018-27093-x (PMC5993796; doi:10.1038/s41598-018-27093-x)
Supplement: Supplementary file 1 — Enhanced quantification of metabolic activity for individual adipocytes by label-free FLIM [file 41598_2018_27093_MOESM1_ESM.pdf]

Title: Enhanced quantification of metabolic activity for individual adipocytes by label-free FLIM

Authors: Michael Evers[1,2], Nunciada Salma[1], Sam Osseiran[3], Malte Casper[1,2], Reginald Birngruber[2], Conor L. Evans[3] & Dieter Manstein[1]

[1]Cutaneous Biology Research Center, Department of Dermatology, Massachusetts General Hospital, Harvard Medical School, Boston, MA, 02129, USA.
[2]Institute of Biomedical Optics, University of Lübeck, Lübeck, 23562, Germany.
[3]Wellman Center for Photomedicine, Department of Dermatology, Massachusetts General Hospital, Harvard Medical School, Boston, MA, 02114, USA.

Corresponding Author: Michael Evers (email: mevers@mgh.harvard.edu phone:(857) 204-3627) or Dieter Manstein (email: dmanstein@mgh.harvard.edu phone:(617) 726-4893)

149 13th Street, Cutaneous Biology Research Center, Department of Dermatology, Massachusetts General Hospital, Harvard Medical School, Boston, MA, 02129, USA.

Keywords: FLIM, NADH, Phasor, Metabolism, Multi-photon

Supplementary Information

Supplementary Note 1. Damage Threshold

Studies by Hopt and Neher on cellular photo-damage of mammalian cells induced by ultrafast near-infrared laser sources under typical *in vitro* imaging conditions, revealed that the number of scans before photo damage occurs is empirically given by [1]:

$$m = \frac{f^{1.5} * \lambda^2 * \tau^{1.5}}{t_{dwell} * A * NA * n * \langle P(t) \rangle^{2.5}} * \frac{1}{Cn_1 * Cn_2 * Cn_3 * Cn_4} \quad (1)$$

	Symbol	Hopt	König	Nan	Evers
Pulse width [fs]	τ	190	150	2000	120
Wavelength [nm]	λ	840	800	711	755
Power [mW]	*P(t)*	10	7.1	63	11.25
Frequency [MHz]	*f*	82	80	80	80
Dwell time [µs/pixel]	t_{dwell}	10	80	3.3	2
Area of a cell [pixel]	*A*	1500	1500	4625	3250
Numerical Aperture	*NA*	0.9	1.25	1.2	1.2
Refractive Index	*n*	1.33	1.33	1.33	1.33
Constant 1 [$m^2/mW^{2.5}s$]	Cn_1	$4.5 * 10^{-23}$			
Constant 2	Cn_2	$\pi^{2.5}$			
Constant 3	Cn_3	0.216			
Constant 4	Cn_4	0.576			
Damage Threshold Scan Number	*m*	75	10	10	40

Supplementary Table 1: Shows the laser settings used during the experiments and the maximum number of scans to reach the damage threshold.

The damage threshold proposed by Hopt and Neher observed in Chinese hamster ovary cells was empirically determined using both abrupt changes in basal [Ca^{2+}], as measured by the fluorescence indicator dye FURA-2, and morphological changes. Other experiments determining the damage threshold were carried out by König *et al.*, observing the cloning efficiency of Chinese hamster ovary cells after exposure to laser irradiation in a two-photon microscope. Nan et al., for their part, evaluated laser-induced effects based on changes of cellular morphology such as breakdown of the cytoskeleton or extracellular matrix (ECM), seen as a collapse of the cell body or cell movements [2,3]. These experiments indicated that photo-damage is a nonlinear function of the input beam, underlining that higher order light-matter interactions contribute to the damaging process. Above the threshold, two-photon photochemical photodamage as well as immediate cell damage via optical breakdown may occur. Comparing the numbers of these previous experiments with our excitation conditions, using Eq. 1, we find a tolerable number of 40 scans for an average illumination power of 11.25 mW. In order to generate good enough photon statistics for low spatial binning, 160 scans at 11.25 mW power were used in our experiments which is 4-times the number of scans proposed by the damage threshold. The fact that we didn't observe cellular responses such as photobleaching, change of morphology or increased rate of apoptosis to laser radiation at these laser parameters and high scan numbers might be explained by the choice of cells as well as excitation wavelength. Indeed, it is worth noting that the experiments by Hopt and Neher, as well as by König et al., were carried out on Chinese hamster ovary cells, which complicate a direct comparison with our results. Nan et al., on the other hand, used mouse adrenal cortical tumor (Y-1) and fibroblast cells (3T3-L1, ATCC), which are more similar to the cells used in our study.

However, the experiments carried out by Nan et al. used a laser excitation wavelength of 711 nm compared to 755 nm in our study. Patterson et al. have reported that when the excitation wavelength for NADH was increased from a high two-photon cross section (710 nm) to a low two-photon cross section (760 nm) with matched intensities to yield similar photon excitation, the low two-photon cross section wavelengths had 3-fold lower photobleaching rates [4]. These findings support our observation of the absence of cellular photo-damage after laser irradiation that exceeds the empirical damage threshold by a factor of 4.

Supplementary Note 2. Apoptosis

The detection of laser induced cellular damage leading to programmed cell death (apoptosis) is of utmost importance in FLIM studies. Caspases are signaling molecules that are markers for cellular damage. Although the precise role in the initiation and progression of apoptosis is not yet known for all caspases, their use for the detection of apoptotic events it is commonly used as a readout in apoptosis assays. 3T3-L1 adipocytes were stained with CellEvent™ Caspase-3/7 Green Detection Reagent (ThermoFisher) as per the manufacturer's protocol. A trans-illumination image of the 3T3-L1 fat cells in combination with an apoptosis fluorescence image were taken, prior to a 60 s FLIM image with the experimental laser settings at 755 nm wavelength and 11.25 mW power. Another trans-illumination image in combination with an apoptosis fluorescence image were taken 4 hours after the FLIM image to see if apoptosis was induced by the laser irradiation. Given the high degree of similarity between the two imaged timepoints and the relatively constant signal intensity from the fluorescent apoptosis marker, we can safely conclude that the imaging conditions do not induce apoptosis.

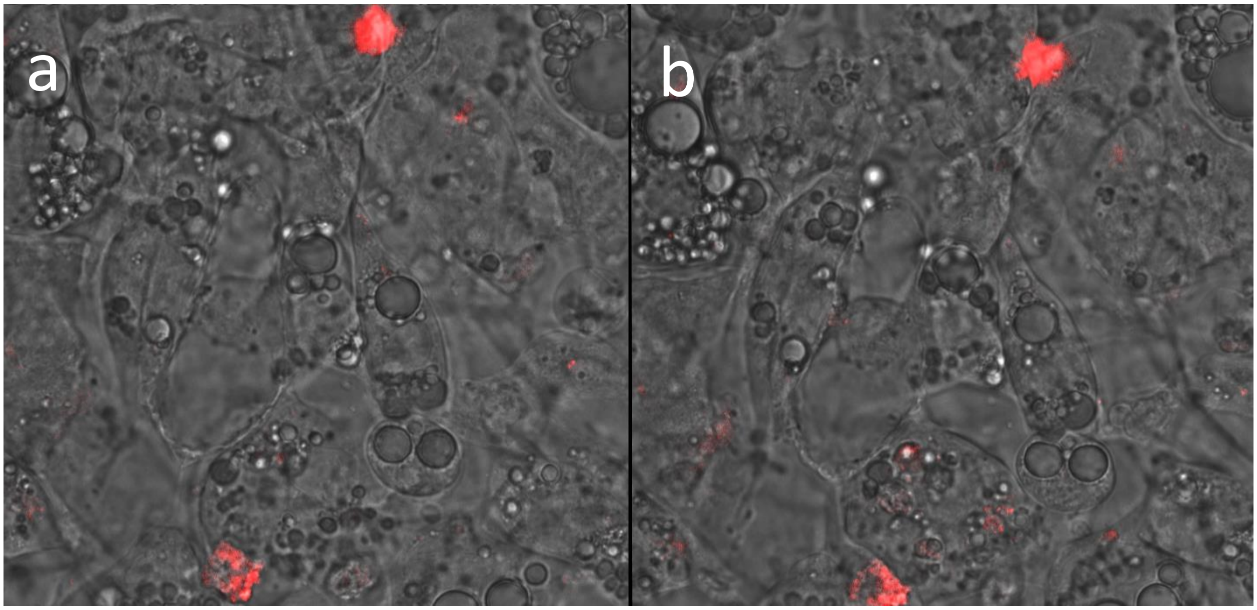

Supplementary Figure 1: Apoptosis stain images. (**a**) Trans-illumination image of 3T3-L1 fat cells in combination with a fluorescence image. Fluorescence is shown in red. After this image was captured a FLIM image was taken. (**b**) Image of the same region 4 hours after the FLIM image was taken.

Supplementary Note 3. NADH Solution Measurements

Pure NADH in solution and a mixture of NADH and LDH (16:1) in solution were analyzed. For both solutions, a double and a triple exponential fit of the fluorescence decay curve were used to find the appropriate model to generate accurate fluorescence lifetimes. The double exponential fit of NADH resulted in a short fluorescence lifetime component at 0.4 ns and a long fluorescence lifetime component at 0.9 ns which agree well with literature and stand for a possibly folded (0.4 ns) and extended (0.9 ns) conformation of free NADH [5,6]. A triple-exponential fit of pure NADH in solution resulted in over-fitting, which can be identified by two almost identical lifetime components with lifetimes of 0.4 ns and 0.5 ns. The double-exponential fitting of the NADH-LDH mixture resulted in a significant increase of lifetime for the long lifetime component to 1.7 ns compared to pure NADHs long lifetime component at 0.9 ns. The triple-exponential fit of the NADH-LDH solution introduced a third lifetime component at 2.1 ns additionally to the two NADH lifetime components from pure NADH in solution. We therefore assume that the double-exponential fit for pure NADH and the triple-exponential fit for the NADH-LDH mix are the appropriate models for the fluorescence lifetime analysis. The third lifetime component of the NADH-LDH mix at 2.1 ns represents the fluorescence lifetime of LDH-bound NADH.

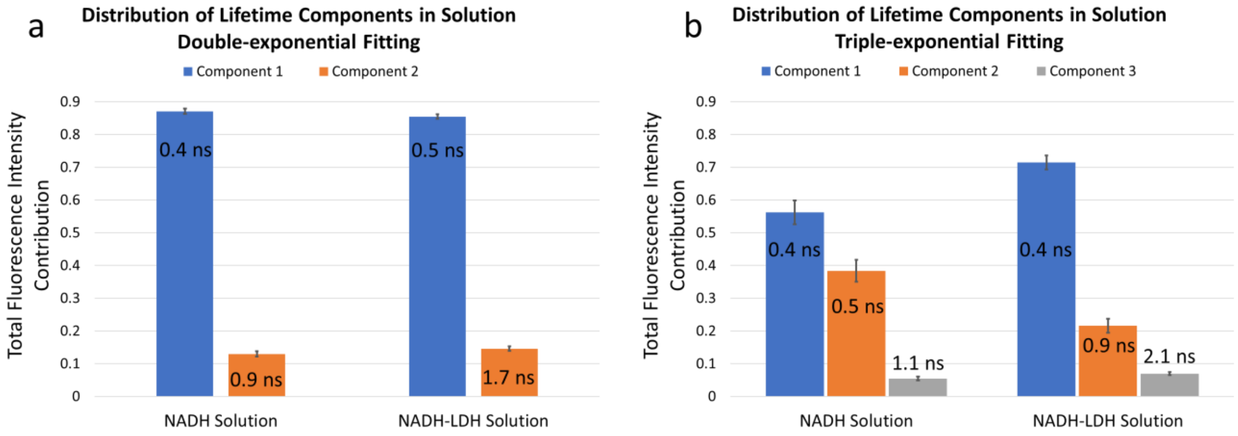

Supplementary Figure 2: Double-, and triple-exponential fluorescence lifetime decay fittings (**a**) double-exponential fitting of NADH and a mixture of NADH and LDH (16:1) in solution (**b**) triple-exponential fitting of NADH and a mixture of NADH and LDH (16:1) in solution

Supplementary Note 4. Mitochondria Movement

Multiple fluorescence images of 3T3-L1 fat cells using a mitochondrial stain were captured 6 s apart from each other to analyze the movement of individual mitochondria. Images taken 6 s apart from each other showed considerable mitochondrial movement. While the movement analysis in areas where the mitochondria were clustered within the cell was not feasible, less clustered areas were used to measure the displacement between frames. Image analysis revealed an average displacement of 0.5 µm movement within 6 s in less clustered areas of the cell. During the acquisition time of a FLIM image using the experimental setup (60 s) mitochondria were found to move up to 5 µm. The FLIM imaging system uses a 60x objective lens with 1.2 NA to excite very small focal volumes. Given the natural intracellular migration of mitochondria during the 60 s acquisition period, mitochondria inevitably enter and exit the focal plane throughout acquisition, leading to image blurriness as shown in supplementary figure 5 and thus having negative impact on the fluorescence lifetime analysis. While there are areas of the cell that only exhibit fluorescence of mitochondria or the cytoplasm during the acquisition time, there are also areas that display a mixture of cytosolic and mitochondrial fluorescence. The double-exponential model used in this work is not suited for these mixtures of cytosolic and mitochondrial fluorescence, which leads to inaccurate fluorescence lifetimes. This inaccuracy induced by the mitochondria movement broadens the peaks of the individual lifetime components and makes it difficult to separate each component individually.

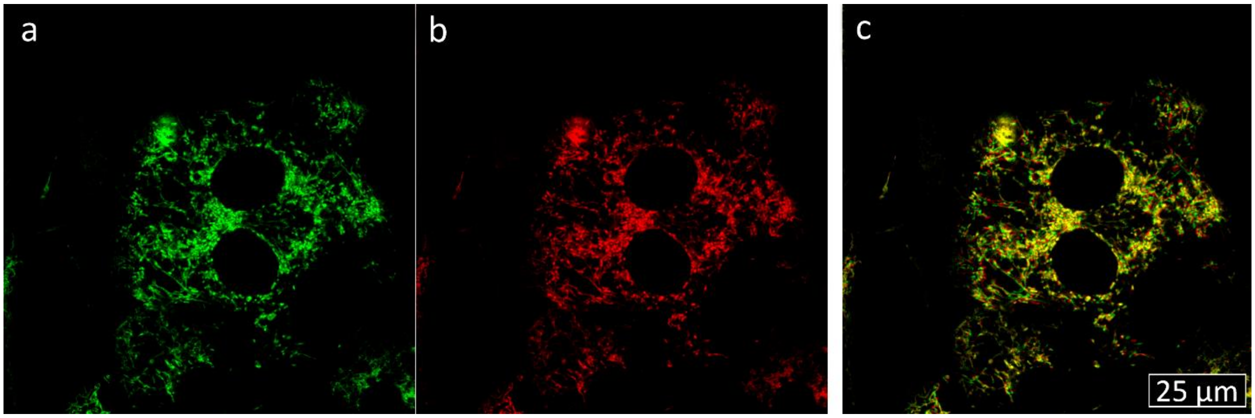

Supplementary Figure 3: Mitochondrial stain images showing the movement of mitochondria. (**a,b**) Mitochondrial stain image of a single 3T3-L1 fat cell captured 6 second apart from each other. (**c**) Combination of image a and b to show the movement. In areas where the mitochondria are clustered it is not feasible to detect the displacement and a yellow color is visible (additive of green and red).

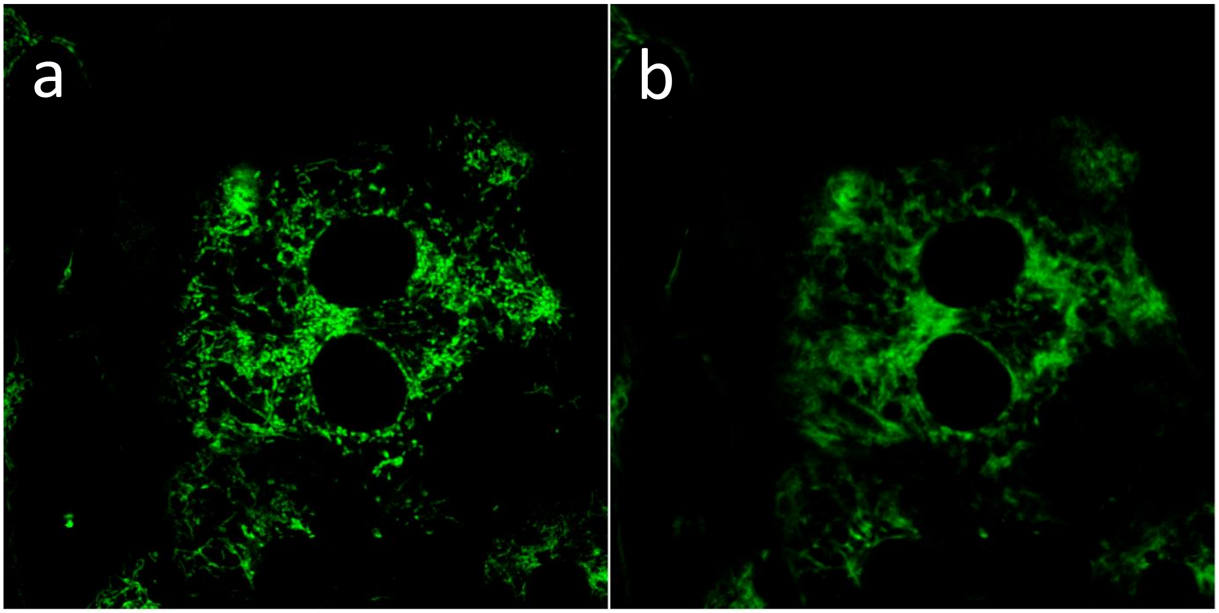

Supplementary Figure 4: Mitochondrial stain image of single 3T3-L1 fat cell. (**a**) Single image taken within 6 seconds (**b**) Sum of 10 images taken over a period of 60 seconds.

Supplementary Note 5. Lifetime Fitting

It is often not clear which model, in particular which number of exponential components, should be used to fit the data. In literature, endogenous fluorescence of NADH in live cells has been fitted with double, triple and quadruple-exponential fits [5–8]. One way to verify model accuracy is by fitting the decay curve with a varying number of exponential components, and comparing the chi-squared (χ^2) value as well as the residuals, which represent the goodness of fit. A good fit is characterized by a χ^2 close to 1, and residuals showing no noticeable systematic variations [9]. If more exponential components are defined than needed to fit the data, the fitting returns two components of almost identical lifetime, or an extremely long lifetime component of very low relative amplitude. Most often, fitting data with a minimal number of components delivers acceptable χ^2 values, while adding supplemental exponential components only results in minor improvements in fit. This is another indication that the model cannot be made more accurate by adding exponential components. Supplementary figure 5 shows that the single exponential fit results in poor χ^2, which is markedly improved by using a double exponential fit. Even though a triple exponential fit further improves the χ^2 value, the additional lifetime component τ_3 is almost identical to lifetime component τ_2, which suggests overfitting.

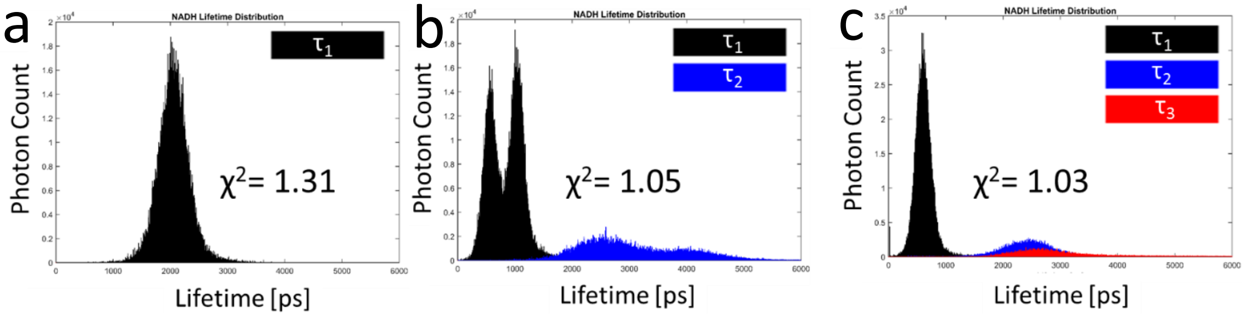

Supplementary Figure 5: Using single-, double-, and triple-exponential fits on the same data set of NADH fluorescence of adipocytes to determine the correct fitting model. (**a**) An attempt to fit the data with a single exponential results poor χ^2 values. (**b**) Double exponential fitting results in good χ^2 values. (**c**) Triple exponential fitting delivers a small in improvement in χ^2 values, but the third lifetime component τ3 is almost identical to lifetime component τ2, which suggests overfitting.

Supplementary Note 6. pH-Values after Injection

The pH of a solution is known to impact the fluorescence lifetime of a dissolved fluorophore [10]. Fluorescence lifetimes of adipocytes' NADH changed significantly after the injection of several pharmacological reagents. To confirm whether the fluorescence lifetime change was indeed based on metabolic changes rather than change in extracellular pH, media acidity was monitored using a pH meter. Individual injections of the pharmacological reagents were performed in the same concentration and media as in cell experiments. We were able to show that none of the reagents at given concentration resulted in a significant change in pH.

Solution and Reagents	Average pH Value	Standard Deviation
Glycolysis Stress Test Media (2 mM glutamine)	7.02	0.03
Glucose (10mM)	7.05	0.02
Oligomycin (1 µM)	7.07	0.03
2-DG (100 mM)	7.06	0.02
Forskolin (5 µM)	7.07	0.02
Epinephrine (0.5 µM)	7.09	0.04
Mitochondria Stress Test Media (10 mM glucose, 1 mM pyruvate, 2 mM glutamine)	7.08	0.02
Oligomycin (1 µM)	7.16	0.04
FCCP (1 µM)	7.15	0.05
Rotenone AA (0.5 µM)	7.14	0.06
Forskolin (5 µM)	7.17	0.03
Epinephrine (0.5 µM)	7.19	0.05

Supplementary Table 2: Shows pH-value changes of the glycolysis and mitochondria stress test media after injection of pharmacological reagents.

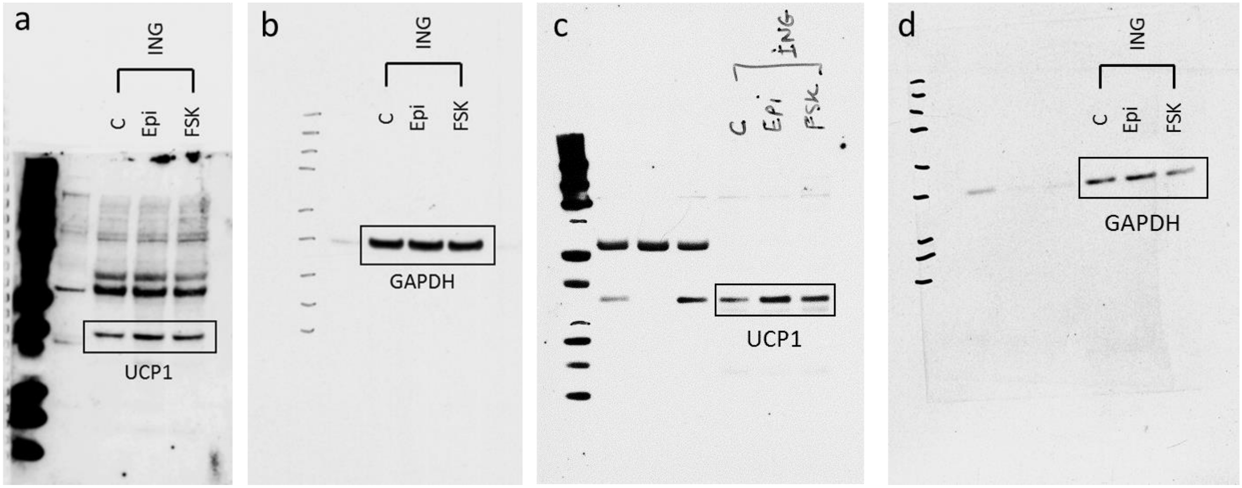

Supplementary Figure 6: Complete western blots showing the effect of epinephrine and forskolin on the expression of UCP1 and GAPDH of murine inguinal white fat.

Supplementary Note 7. Western Blots - complete blots

Literature shows that thermogenesis of brown fat can be initiated through mild cold application or through pharmacological reagents [11]. It was also demonstrated that mitochondria of inguinal white fat have sufficient UCP1 protein levels to activate thermogenesis [11]. Supplementary figure 6 shows western blots of UCP1 gene expression of inguinal white fat without treatment and with treatment of epinephrine and forskolin. Untreated inguinal fat cells initially express low levels of UCP1, while epinephrine and forskolin treated cells show an increased UCP1 expression. Epinephrine and forskolin promote lipolysis and are known to induce browning. UCP1 expression was normalized to the expression of reference gene GAPDH which remained constant under investigation for all three groups.

Supplementary Note 8. Photobleaching

Photobleaching experiments were carried out on 3T3-L1 adipocytes using four different powers in the focal plane for continuous measurement over 15 minutes. During this experiment 60 second FLIM images were taken continuously for 15 minutes and the total intensity of the field of view was analyzed for each image. While the intensity for powers of 11.25 mW didn't show significant

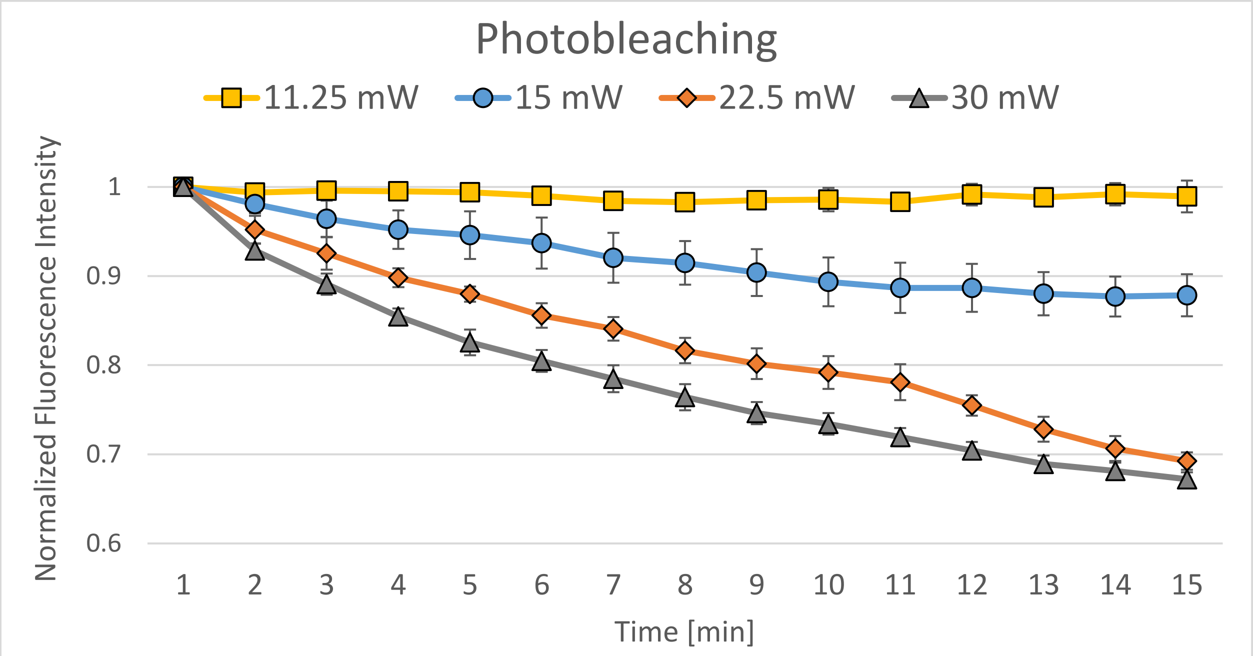

Supplementary Figure 7: Shows the change of fluorescence intensity of 3T3-L1 adipocytes over a 15-minute duration using different laser excitation powers at the focal plane at 755 nm wavelength.

changes over the 15-minute duration, the fluorescence intensity decreased for powers of 15mW and higher. It appears that the cells can compensate for photobleaching at low laser intensities but after many frames or at higher intensities, the cells become unable to adapt.

Supplementary Note 9. Multi-harmonic phasor analysis

A common way to separate components having the same phasor location but different lifetime distributions is to perform a multi-harmonic analysis of the fluorescence lifetime data with higher harmonics of the laser repetition rate, wherein the harmonics are $\omega = n\omega_o$ with n=2, 3 [12]. The idea of this method is that the phasor histogram does not broaden very much as the frequency is increased and that the phasor histogram shifts along the universal circle toward the (0,0) coordinate for increasing frequencies. The best frequency for maximal sensitivity to changes in the phasor location is when the phasor is located in the central region of the phasor plot. If the repetition rate is too high, the phasors of various molecular species will be crowed in a small area of the phasor plot, and their separation becomes problematic. Analyzing the phasor plots of NADH of adipocytes at higher harmonics (2 and 3) of the laser repetition rate did not result in a separation of mitochondrial and cytosolic NADH populations (supplementary figure 9). This inability of further separating the phasor points was expected since the phasor cluster of the first harmonic already had a central position within the semicircle which results in maximum sensitivity.

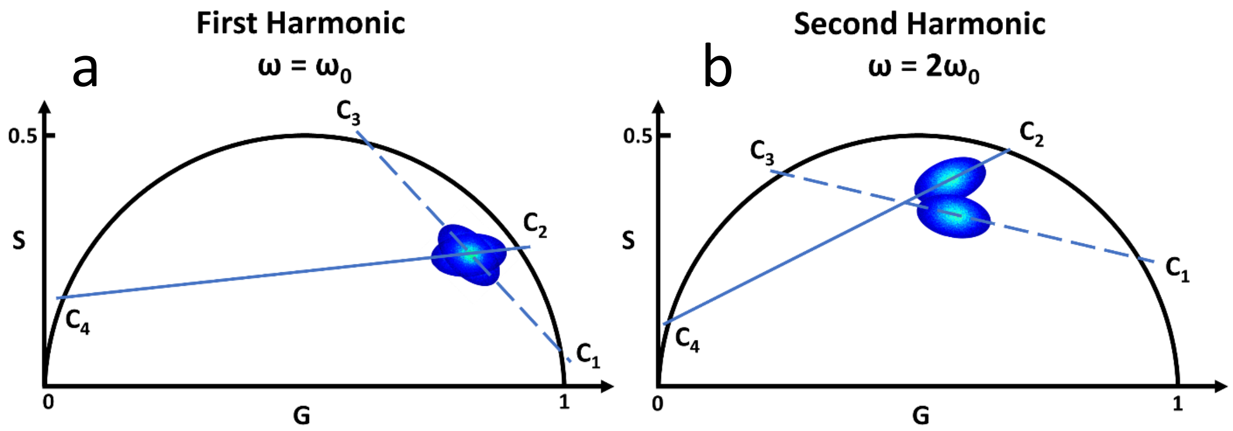

Supplementary Figure 8: Scheme showing that the phasor analysis can be performed in any harmonic of the original modulation frequency. If two phasors are the linear combination of a different pair of phasors, for example C_1-C_3 and C_2-C_4, they may overlap in the first harmonic (**a**), but may be separated at higher harmonic (**b**) [11]

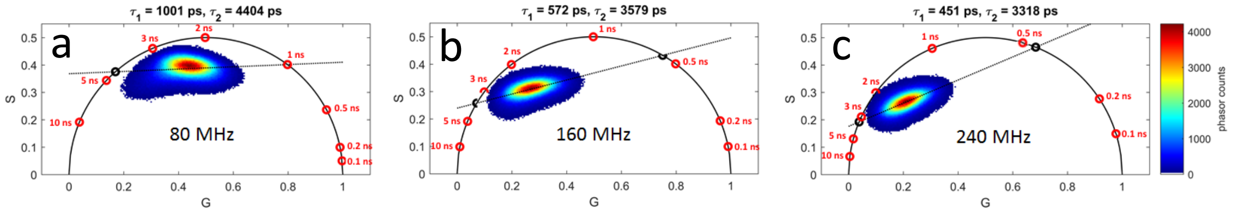

Supplementary Figure 9: Showing the phasor analysis of NADH of adipocytes at different harmonics of the laser repetition rate. (**a**) first harmonic of the original modulation frequency at 80 MHz (**b**) second harmonic at 160 MHz (c) third harmonic at 240 MHz

Supplementary Note 10. Fluorescence lifetime analysis of isolated mitochondria

To show regional differences of the NADH species, mitochondria of 3T3-L1 adipocytes were isolated. The isolated mitochondria were stained with TMRE to check for purity. Even though some impurities were detected (supplementary figure 10) most of the fluorescence signal originated from mitochondria. As expected, mitochondria predominately showed fluorescence from lifetime component C_1 and C_3. This finding strengthens the claim that the fluorescence lifetime is specific to cellular features.

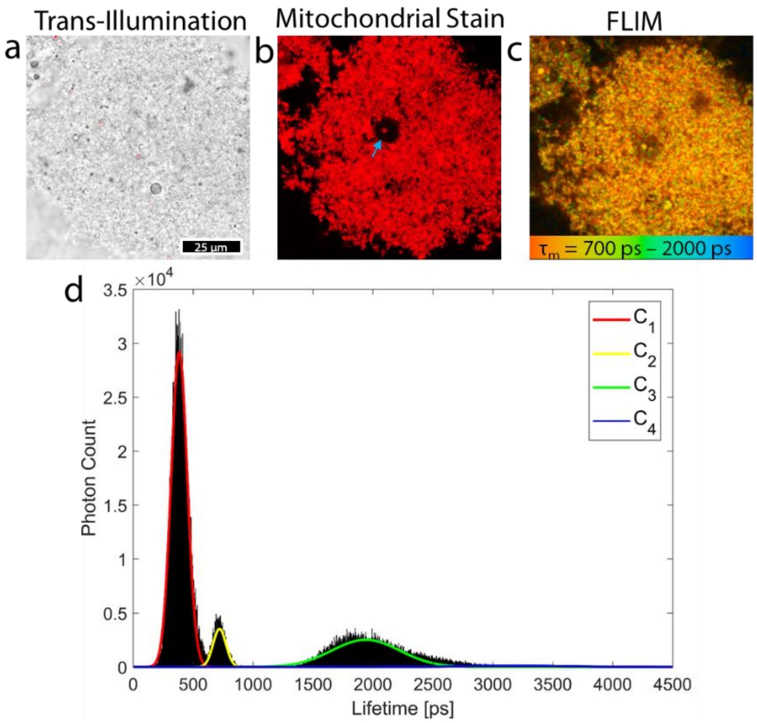

Supplementary Figure 10: Fluorescence lifetime distribution of NADH of isolated mitochondria of 3T3-L1 adipocytes. (**a**) Trans-illumination image, (**b**) TMRE mitochondrial stain image, and (**c**) FLIM image of isolated mitochondria. The blue arrow in the mitochondrial stain image shows a cell nucleus. (**d**) Fluorescence lifetime distribution of isolated mitochondria.

Supplementary Note 11. Redox Ratio

The redox ratio is originating from two fluorescent cofactors: NADH and FAD. Both molecules play crucial roles in electron transport in the cell and changes in their concentration can be used to determine the metabolic state of a cell. The relative fluorescence intensity of NADH and FAD can be expressed as FAD^+ / (FAD^+ + NADH), namely the optical redox ratio. Literature shows that an increase in redox ratio values are linked to oxidative phosphorylation while a decrease in redox ratio means an increase in glycolytic metabolism. Supplementary Figure 11 and 12 show changes of NADH intensity, FAD^+ intensity, and redox ratio after individual injection of several pharmacological reagents of the mitochondria stress test and glycolysis stress test. **Our**

results conform with other reports showing significant variations in redox ratio due pharmacologically induced cellular metabolism. [13,14]

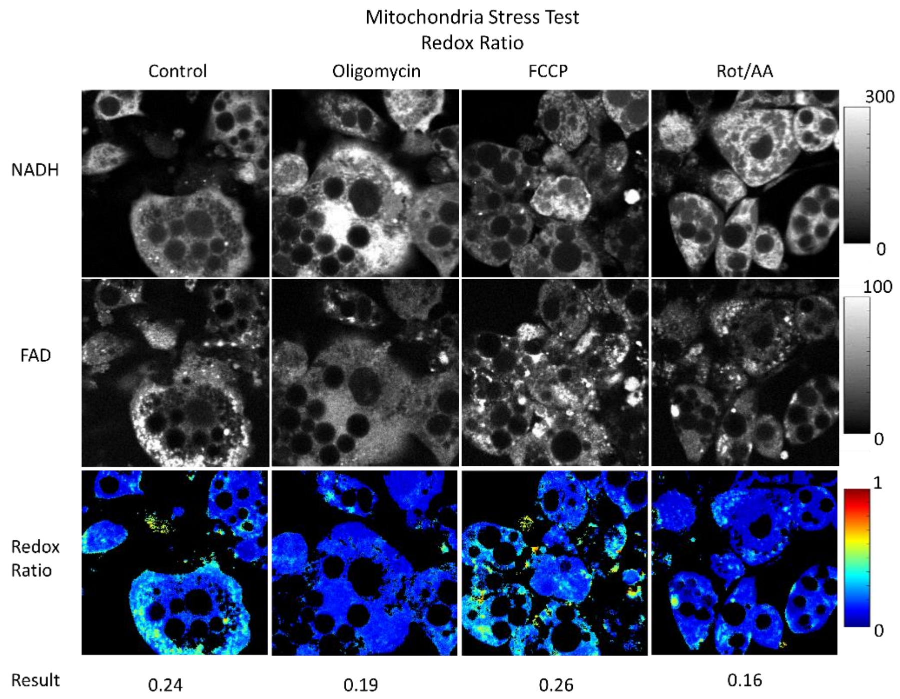

Supplementary Figure 11: Redox ratio based on the fluorescence intensity of NADH and FAD for individual injections of the mitochondria stress test. Representative images of NADH, FAD and the calculated redox ratio (FAD/FAD + NADH) in response to individual injections of mitochondrial inhibitors and uncouplers. All images were acquired from different cell plates of the same culture under identical conditions after individual injection of each drug indicated above the figure panels. The last row shows the average value of the redox ratio.

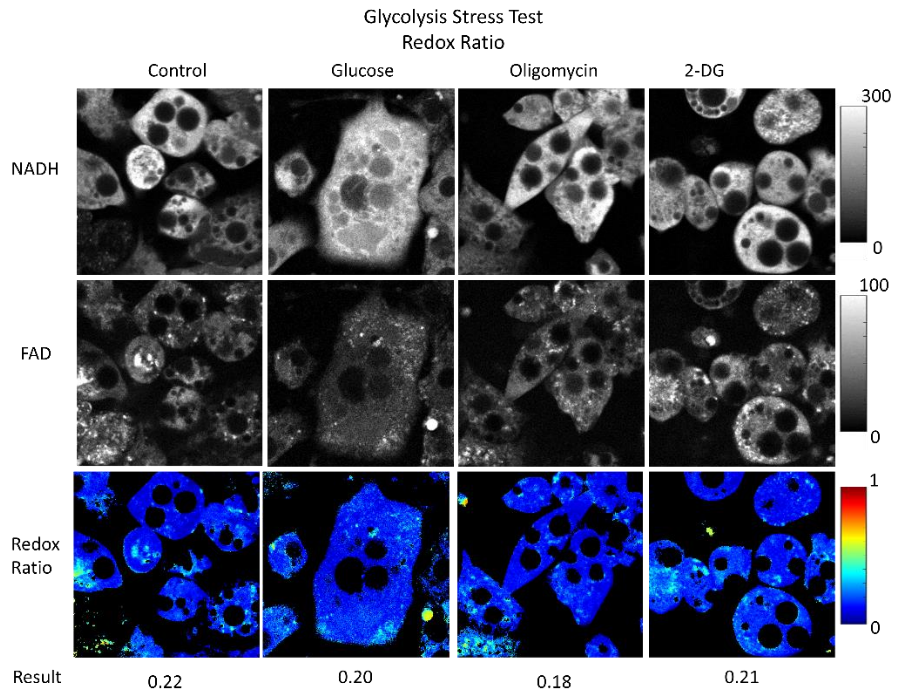

Supplementary Figure 12: Redox ratio based on the fluorescence intensity of NADH and FAD for individual injections of the glycolysis stress test. Representative images of NADH, FAD and the calculated redox ratio (FAD/FAD + NADH) in response to individual injections of drugs changing

the glycolic rate. All images were acquired from different cell plates of the same culture under identical conditions after individual injection of each drug indicated above the figure panels. The last row shows the average value of the redox ratio.

Supplementary Note 12. Detection of Lipids and Lipofuscin

Lipofuscin is mainly present in post mitotic cells. It consists of lipids, proteins, carbohydrates and a small amount of metals. Lipofuscin has been investigated concerning its lipid and protein composition and several authors described a protein fraction of 20 to 70% and a lipid fraction of 20 to 50 wt%.[15,16] Stimulated Raman Scattering (SRS) microscopy is highly sensitive to the detection of lipids. Due to the lipid rich composition of lipofuscin, this imaging modality is able to reliably detect lipofuscin.[17] The contrast of the SRS signal in the lipid droplets arises from the Raman response of the abundant C-H bonds in the lipid molecules.[18] Thus, laser scanning Raman scattering microscopy is applied to visualize lipid droplets in cells and tissues. There are several publications using fluorescent dyes for labeling of lysosomes and lipids such as LysoTracker to detect lipofuscin.[19,20] Colocalization studies using Raman scattering microscopy showed high correlation with these fluorescent dyes for imaging of lipid and lysosome-related organelles.[20–22] Therefore, SRS was used to detect lipofuscin and other lipid-rich components in Supplementary Figure 13. To eliminate the influence of lipid and lipofuscin fluorescence on the analysis of NADH fluorescence, regions containing lipid

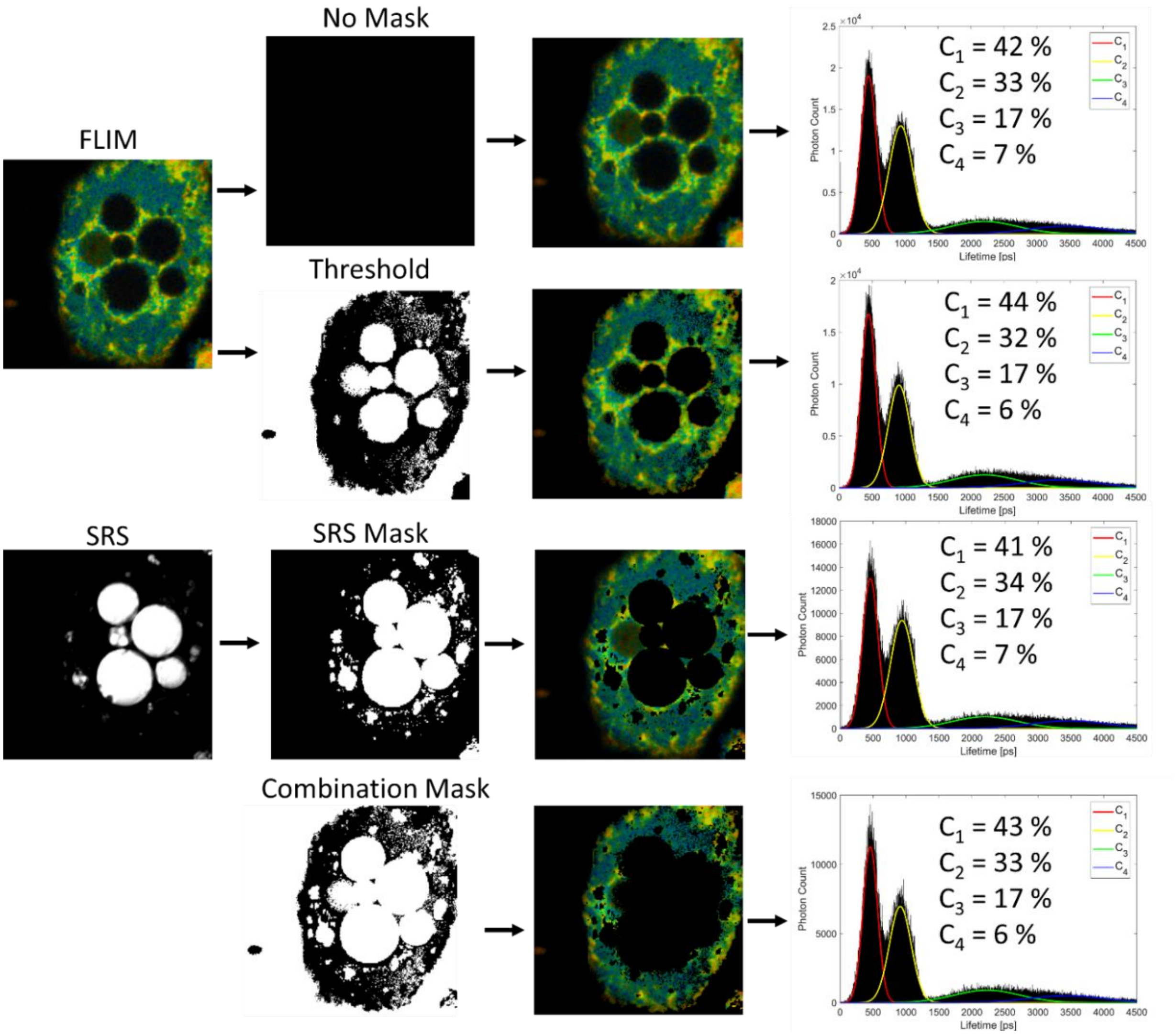

Supplementary Figure 13: FLIM, CARS and SRS image of a single 3T3-L1 adipocyte. An intensity based adaptive threshold was used to create a mask to remove low fluorescence intensity areas such as the background, nuclei, and lipid droplets. CARS and SRS signals were used to generate masks containing lipids and lipofuscin. The last row shows a combination masks generated by addition of all other masks. The masks were used to removed certain features of the FLIM image for the fluorescence lifetime analysis and the resulting lifetime distributions are shown in the column on the right.

and lipofuscin were removed from the analysis. These regions were determined by SRS and fluorescence intensity as shown in Supplementary Figure 13. While initially analyzing the entire image, several masks were applied such as an intensity based adaptive threshold mask, a mask based on the SRS signal of lipids and a combination of all the masks. The analysis of the fluorescence lifetime distribution after application of these masks, which eliminate the fluorescence from lipids and therefore also from lipofuscin, resulted in 4 distinct lifetime components for NADH. This shows that all 4 lifetime components are indeed part of the fluorescence of NADH.

Supplementary References

1. Hopt, A. & Neher, E. Highly nonlinear photodamage in two-photon fluorescence microscopy. *Biophys. J.* **80,** 2029–2036 (2001).
2. König, K. Multiphoton microscopy in life sciences. *J. Microsc.* **200,** 83–104 (2000).
3. Nan, X., Potma, E. O. & Xie, X. S. Nonperturbative Chemical Imaging of Organelle Transport in Living Cells with Coherent Anti-Stokes Raman Scattering Microscopy. *Biophys. J.* **91,** 728–735 (2006).
4. Patterson, G. H. & Piston, D. W. Photobleaching in two-photon excitation microscopy. *Biophys. J.* **78,** 2159–2162 (2000).
5. Vergen, J. *et al.* Metabolic Imaging Using Two-Photon Excited NADH Intensity and Fluorescence Lifetime Imaging. *Microsc. Microanal.* **18,** 761–770 (2012).
6. Yaseen, M. A. *et al.* In vivo imaging of cerebral energy metabolism with two-photon fluorescence lifetime microscopy of NADH. *Biomed. Opt. Express* **4,** 307 (2013).
7. Meleshina, A. V. *et al.* Probing metabolic states of differentiating stem cells using two-photon FLIM. *Sci. Rep.* **6,** 21853 (2016).
8. Alonzo, C. A. *et al.* Two-photon excited fluorescence of intrinsic fluorophores enables label-free assessment of adipose tissue function. *Sci. Rep.* **6,** 31012 (2016).
9. Wolfgang Becker. *The bh TCSPC Handbook*. (Becker & Hickl GmbH, 2014).
10. Islam, M. S., Honma, M., Nakabayashi, T., Kinjo, M. & Ohta, N. pH Dependence of the Fluorescence Lifetime of FAD in Solution and in Cells. *Int. J. Mol. Sci.* **14,** 1952–1963 (2013).
11. Shabalina, I. G. *et al.* UCP1 in Brite/Beige Adipose Tissue Mitochondria Is Functionally Thermogenic. *Cell Rep.* **5,** 1196–1203 (2013).
12. Sanchez, S., Bakás, L., Gratton, E. & Herlax, V. Alpha Hemolysin Induces an Increase of Erythrocytes Calcium: A FLIM 2-Photon Phasor Analysis Approach. *PLoS ONE* **6,** e21127 (2011).
13. Alhallak, K., Rebello, L. G., Muldoon, T. J., Quinn, K. P. & Rajaram, N. Optical redox ratio identifies metastatic potential-dependent changes in breast cancer cell metabolism. *Biomed. Opt. Express* **7,** 4364–4374 (2016).
14. Hou, J. *et al.* Correlating two-photon excited fluorescence imaging of breast cancer cellular redox state with seahorse flux analysis of normalized cellular oxygen consumption. *J. Biomed. Opt.* **21,** (2016).
15. Brunk, U. T. & Terman, A. Lipofuscin: mechanisms of age-related accumulation and influence on cell function12 1Guest Editor: Rajindar S. Sohal 2This article is part of a series of reviews on “Oxidative Stress and Aging.” The full list of papers may be found on the homepage of the journal. *Free Radic. Biol. Med.* **33,** 611–619 (2002).
16. Warburton, S. *et al. Examining the proteins of functional retinal lipofuscin using proteomic analysis as a guide for understanding its origin*. **11,** (2005).
17. Datta, R., Alfonso-García, A., Cinco, R. & Gratton, E. Fluorescence lifetime imaging of endogenous biomarker of oxidative stress. *Sci. Rep.* **5,** (2015).
18. Nan, X., Cheng, J.-X. & Xie, X. S. Vibrational imaging of lipid droplets in live fibroblast cells with coherent anti-Stokes Raman scattering microscopy. *J. Lipid Res.* **44,** 2202–2208 (2003).
19. Sparrow, J. R., Parish, C. A., Hashimoto, M. & Nakanishi, K. A2E, a Lipofuscin Fluorophore, in Human Retinal Pigmented Epithelial Cells in Culture. *Invest. Ophthalmol. Vis. Sci.* **40,** 2988–2995 (1999).
20. Rice, W., Kaplan, D. & Georgakoudi, I. Two-Photon Microscopy for Non-Invasive, Quantitative Monitoring of Stem Cell Differentiation. *PloS One* **5,** e10075 (2010).
21. Klapper, M. *et al.* Fluorescence-based fixative and vital staining of lipid droplets in Caenorhabditis elegans reveal fat stores using microscopy and flow cytometry approaches. *J. Lipid Res.* **52,** 1281–1293 (2011).
22. Yen, K. *et al.* A Comparative Study of Fat Storage Quantitation in Nematode Caenorhabditis elegans Using Label and Label-Free Methods. *PLOS ONE* **5,** e12810 (2010).
